# Phloroglucinol Oligomers from *Callistemon rigidus* as Novel Anti-Hantavirus Replication Agents

**DOI:** 10.3390/v17070916

**Published:** 2025-06-27

**Authors:** Jin-Xuan Yang, E-E Luo, Yue-Chun Wu, Kai Zhao, Wei Hou, Mu-Yuan Yu, Xu-Jie Qin, Xing-Lou Yang

**Affiliations:** 1 Key Laboratory of Genetic Evolution & Animal Models, Yunnan International Joint Laboratory of Zoonotic Viruses, Yunnan Key Laboratory of Biodiversity Information, Kunming Institute of Zoology, Chinese Academy of Sciences, Kunming 650201, China; yangjinxuan260@163.com (J.-X.Y.); wuyuechun@mail.kiz.ac.cn (Y.-C.W.); zhaokai@mail.kiz.ac.cn (K.Z.); 2 State Key Laboratory of Phytochemistry and Natural Medicines, Kunming Institute of Botany, Chinese Academy of Sciences, Kunming 650201, China; luoee@mail.kib.ac.cn; 3 University of Chinese Academy of Sciences, Beijing 100049, China; 4 State Key Laboratory of Virology, Institute of Medical Virology, School of Basic Medical Sciences, Wuhan University, Wuhan 430071, China

**Keywords:** *Callistemon rigidus*, phloroglucinol oligomers, hantavirus, anti-HTNV activity, molecular docking

## Abstract

Zoonotic viral diseases have continued to threaten global public health in recent decades, with rodent-borne viruses being significant contributors. Infection by rodent-carried hantaviruses (HV) can result in hemorrhagic fever with renal syndrome (HFRS) and hantavirus pulmonary syndrome (HPS) in humans, with varying degrees of morbidity and mortality. However, no Food and Drug Administration (FDA) vaccines or therapeutics have been approved for the treatment of these diseases. In an effort to identify antiviral bioactive molecules, we isolated four oligomeric phloroglucinols from *Callistemon rigidus* leaves, including two new phloroglucinol trimers, callistemontrimer A and B, along with two previously characterized phloroglucinol dimers, rhodomyrtosone B and rhodomyrtone. We evaluated the anti-Hantaan virus (HTNV) activity of these compounds. Notably, callistemontrimer A demonstrated higher anti-HTNV activity compared to ribavirin. Mechanistic studies revealed that callistemontrimer A exerted its antiviral effects by inhibiting viral replication, likely through interaction with RNA-dependent RNA polymerase (RdRp) of HTNV, as supported by molecular docking analysis. These results highlight oligomeric phloroglucinols as promising lead candidates for the development of anti-HV therapeutics.

## 1. Introduction

Hantaviruses (HVs) comprise a diverse group of enveloped, negative-sense, single-stranded RNA viruses with tripartite genomes, encompassing both pathogenic and non-pathogenic viruses. Among them, the pathogenic viruses are zoonotic in origin and are responsible for severe human infections worldwide. These viruses are predominantly carried by rodents and can be transmitted to humans, leading to two major clinical syndromes: hantavirus pulmonary syndrome (HPS) in the Americas and hemorrhagic fever with renal syndrome (HFRS) in Asia and Europe [1,2]. HFRS is primarily associated with infections by the Hantan virus (HTNV), Seoul virus (SEOV), Puumala virus (PUUV), Dobrava-Belgrade virus (DOBV), and Amur virus (AMRV). HTNV, the prototype member of this group, was first isolated in 1978 by Li et al. from the striped field mouse (*Apodemus agrarius*)—its natural reservoir—in Hantaan County, Korea, and designated the strain 76-118. HTNV is recognized as the most virulent agent among HFRS-causing HVs. In contrast, HPS is primarily caused by the Sin Nombre virus (SNV) and Andes virus (ANDV) in North and South America, respectively [1,3,4]. Between 2004 and 2019, mainland China reported 209,209 cases of HFRS, resulting in 1855 fatalities [5]. These data underscore the significant morbidity and mortality rates associated with HV infections and highlight their persistent threat to global public health.

Current therapeutic strategies for HV infections remain largely confined to supportive and symptomatic care, with no effective targeted treatments currently available. Although several antiviral agents, including ribavirin, favipiravir, and lactoferrin, have shown potential anti-HV activity in vitro or limited clinical contexts [6,7], their efficacy remains insignificant. In parallel, multiple vaccine platforms, including virus-like particle vaccines, recombinant proteins, viral-vector recombinant vaccines, and nucleic acid-based molecular vaccines, have been explored but have thus far demonstrated limited immunogenicity and insufficient protection in clinical or preclinical settings [8,9]. To the best of our knowledge, no specific antiviral therapies have been approved by the Food and Drug Administration (FDA) for the prevention or treatment of HV infections, highlighting the urgent need for the development of more efficient and less toxic therapeutic options and preventive vaccines.

**Figure 1:**
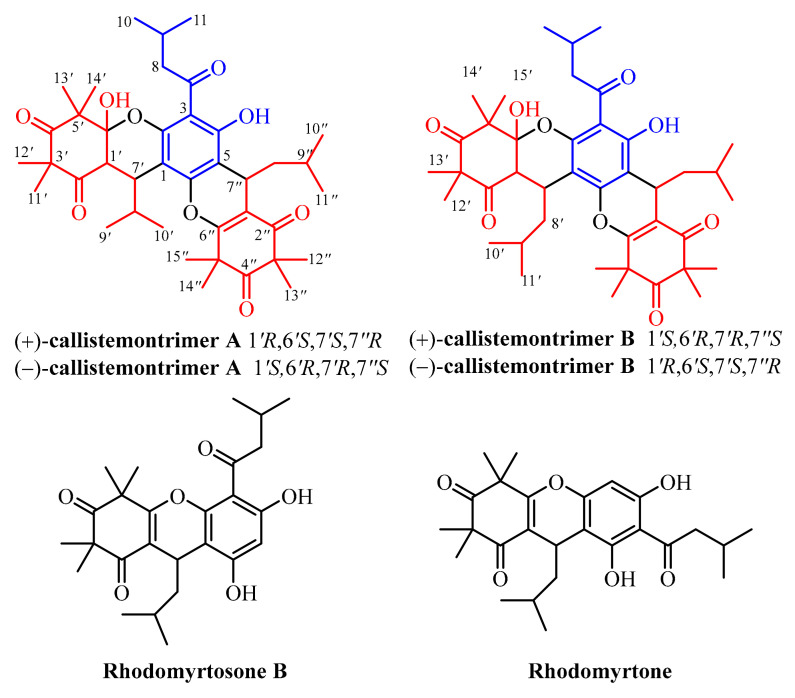
Structures of compounds isolated from *C. rigidus*.

Natural products have historically played a pivotal role in the advancement of new therapeutics. Thus, the exploration of medicinal plants is an effective strategy for the potential discovery of antiviral agents [10,11]. *Callistemon rigidus* R. Br. (Myrtaceae) is a traditional Chinese medicinal plant used to treat conditions such as cough, bronchitis, asthma, pain and swelling from impact injuries, eczema, and rheumatic arthralgia [12]. Widely cultivated in southern China for its ornamental value [13], this species—and the genus *Callistemon* more broadly—is known to produce phloroglucinol derivatives with documented antiviral activity [14]. Building upon our ongoing investigations into bioactive phloroglucinols from Myrtaceae species [15,16,17,18,19], four oligomeric phloroglucinols were successfully isolated from the leaves of *C. rigidus* collected in Yunnan province, China, including two new phloroglucinol trimers, callistemontrimer A and B, together with two previously characterized phloroglucinol dimers (rhodomyrtosone B and rhodomyrtone) (Figure 1). The anti-HTNV activities of these compounds, along with their underlying antiviral mechanisms, were systematically evaluated.

## 2. Materials and Methods

### 2.1. General Experimental Procedures

Ultraviolet (UV) spectra were recorded using a Shimadzu UV2401 PC spectrophotometer (Shimadzu Corporation, Kyoto, Japan). Optical rotations were measured with a JASCO P-1020 automatic digital polarimeter (Rudolph Research Analytical, Easton, PA, USA). Electronic circular dichroism (ECD) spectra were measured using an Applied Photophysics V100 spectropolarimeter (Applied Photophysics, Leatherhead, UK). Nuclear magnetic resonance (NMR) spectra were acquired using a Bruker AVANCE III-600MHz spectrometer (Bruker, Berlin, Germany). High-resolution electrospray ionization mass spectrometry spectra (HRESIMS) were acquired with an Agilent 1290 UPLC/6540 Q-TOF mass spectrometer (Agilent, Santa Clara, CA, USA). Infrared spectroscopy (IR) was measured using a Tensor-27 infrared spectrophotometer with KBr pellets (Bruker, Berlin, Germany). Single-crystal diffractions were collected on a D8 Quest diffractometer using Cu Kα radiation (Bruker, Berlin, Germany). Silica gel (200–300 mesh, Qingdao Marine Chemical Industrials, Qingdao, China) and RP-18 (50 μm, YMC ODS-A-HG, JP) were used for column chromatography (CC). Semi-preparative and chiral high-performance liquid chromatography (HPLC) separations were performed on Agilent 1260 and Agilent 1100 instruments (Agilent, Santa Clara, CA, USA) with an Agilent ZORBAX SB-C18 (9.4 × 250 mm, 5.0 μm) (Agilent, Santa Clara, CA, USA) and a Daicel CHIRALPAK IC (10 × 250 mm, 5.0 μm) column (Tokyo, Japan), respectively.

### 2.2. Plant Material

The leaves of *C. rigidus*, authenticated by Dr. Xu-Jie Qin (Kunming Institute of Botany, Chinese Academy of Sciences), were collected in May 2019 from Chengjiang, Yuxi, Yunnan Province, China. A voucher specimen (KIB-Q-202203) has been deposited at the State Key Laboratory of Phytochemistry and Natural Medicines, Kunming Institute of Botany, Chinese Academy of Sciences (Kunming, China).

### 2.3. Extraction and Isolation

The crude extract of *C. rigidus* leaves was procured by pulverizing dried leaves (4.6 kg) and repeatedly immersing them in petroleum ether. The combined extracts were collected and evaporated under reduced pressure, yielding a concentrated crude extract (70.0 g). The extract was further processed using a MeOH–H_2_O solution (80:20) to produce the requisite separation materials (17.0 g). A silica gel column (70 × 250 mm, 550 g) eluted with a gradient of petroleum ether–ethyl acetate (70:1 → 10:1, *v*/*v*) was applied to separate the extract into five fractions (Fr. A–Fr. E). Fr. E (3.2 g) was subjected to RP-18 silica gel CC with a gradient elution of MeOH–H_2_O (75:25 → 100:0, *v*/*v*), yielding subfractions Fr. E1–Fr. E4. Fr. E3 (0.9 g) was applied to Sephadex LH-20 CC, resulting in the isolation of callistemontrimer A (21.0 mg) as a white powder, which was re-purified via solvent removal. Fr. E4 (1.1 g) was further processed through a silica gel column (35 × 250 mm, 550 g) with a gradient of petroleum ether–chloroform (5:1 → 1:1), followed by semi-preparative HPLC (95:5, MeCN–H_2_O, *v*/*v*), isolating rhodomyrtosone B (15.6 mg, *t*_R_ = 30.0 min) and rhodomyrtone (11.8 mg, *t*_R_ = 37.0 min). Subsequently, gradient elution chromatography with MeOH–H_2_O (10:90 → 100:0, *v*/*v*) was used to separate Fr. C (4.0 g) into four subfractions (Fr. C1–Fr. C4). Fr. C2 (770 mg) was further subjected to silica gel and Sephadex LH-20 CC, followed by semi-preparative HPLC (MeCN–H_2_O, 97:3, *v*/*v*), yielding compound calliste-montrimers B (10.5 mg, t_R_ = 35.8 min). The subsequent separation of callistemontrimer A and B (4 mg each) was achieved via chiral semi-preparative HPLC (n-hexane–2-propanol, 96:4, *v*/*v*), leading to the isolation of (+)-callistemontrimer A (1.4 mg), (−)-callistemontrimer B (1.5 mg), (+)-callistemontrimer A (1.4 mg), and (−)-callistemontrimer B (1.5 mg), respectively.

### 2.4. Spectroscopic Data

*Callistemontrimer A*: Colorless crystals (MeOH); UV (MeOH) *λ*max (log *ε*) 203 (4.11), 221 (4.15), 254 (3.97), 327 (3.35) nm; [α${]}_{D}^{26}$+151.6 (*c* 0.10, MeOH) for (+)-callistemontrimer A; [α${]}_{D}^{26}$−151.8 (*c* 0.10, MeOH) for (−)-callistemontrimer A; ECD (MeOH, Δ*ε*) *λ*max 224 (+12.55), 278 (−1.49), 322 (+1.43) nm for (+)-callistemontrimer A; ECD (MeOH, Δ*ε*) *λ*max 224 (−12.58), 278 (+1.51), 322 (−1.46) nm for (−)-callistemontrimer A; ^13^C and ^1^H NMR data, Table 1; HRESIMS *m*/*z* 679.3834 [M + H]^+^ (calcd for C_40_H_55_O_9_, 679.3841).

*Callistemontrimer B*: Colorless crystals (MeOH); UV (MeOH) *λ*max (log *ε*) 203 (4.11), 221 (4.15), 254 (3.97), 327 (3.35) nm; [α${]}_{D}^{26}$+178.6 (*c* 0.10, MeOH) for (+)-callistemontrimer B; [α${]}_{D}^{26}$−178.6 (*c* 0.10, MeOH) for (−)-callistemontrimer B; ECD (MeOH, Δ*ε*) *λ*max 246 (+56.93), 292 (−5.54) nm for (+)-callistemontrimer B; ECD (MeOH, Δ*ε*) *λ*max 246 (−56.93), 292 (+5.54) nm for (−)-callistemontrimer B; ^13^C and ^1^H NMR data, Table 2; *m*/*z* 693.4000 [M + H]^+^ (calcd for C_41_H_57_O_9_, 693.3997).

Crystal data for *callistemontrimer A*: C_40_H_54_O_9_•CHCl_3_, *M* = 798.20, *a* = 18.8072(8) Å, *b* = 9.8723(4) Å, *c* = 23.8598(10) Å, *α* = 90°, *β* = 109.492(2)°, *γ* = 90°, *V* = 4176.2(3) Å^3^, *T* = 101.(2) K, space group *P*121*/n*1, *Z* = 4, *μ*(Cu Kα) = 2.412 mm^−1^, 38,989 reflections measured, 7927 independent reflections (*R_int_* = 0.0784). Final *R*_1_ value 0.0736 (*I* > 2*σ*(*I*)). Final *wR*(*F*^2^) value 0.2071 (*I* > 2*σ*(*I*)). Final *R*_1_ value 0.0931 (all data). Final *wR*(*F*^2^) value 0.2223 (all data). Goodness of fit on *F*^2^ 1.080.

Crystal data for *callistemontrimer B*: C_41_H_56_O_9_, *M* = 692.85, *a* = 11.5642(8) Å, *b* = 24.0314(17) Å, *c* = 28.147(2) Å, *α* = 90°, *β* = 93.029(4)°, *γ* = 90°, *V* = 7811.2(10) Å^3^, *T* = 100.(2) K, space group *P*121*/n*1, *Z* = 8, *μ*(Cu Kα) = 0.661 mm^−1^, 70,114 reflections measured, 14,810 independent reflections (*R_int_* = 0.1568). Final *R*_1_ value 0.1449 (*I* > 2*σ*(*I*)). Final *wR*(*F*^2^) value 0.3846 (*I* > 2*σ*(*I*)). Final *R*_1_ value 0.1751 (all data). Final *wR*(*F*^2^) value 0.4040 (all data). Goodness of fit on *F*^2^ 1.063

### 2.5. ECD Calculations

ECD spectra for callistemontrimer A and B were computed using the Gaussian 16 software package. Conformational analyses were carried out using CONFLEX v9.2 software with MMFF94S force fields to give the key conformers within a relative energy of 1.0 kcal/mol. The obtained conformations were optimized at the b3lyp/6-31+g (d) level. The ECD calculations of the optimized conformers were conducted based on TDDFT at the b3lyp/6-311++g (2d,p) level in MeOH using the CPCM model.

### 2.6. Cell Lines and Virus

Vero E6 (ATCC: CRL-1587) cell lines were purchased from the National Collection of Authenticated Cell Cultures. Cells were cultured in Dulbecco’s modified Eagle medium (DMEM) (Thermo Fisher Scientific and Gibco, Waltham, MA, USA; Cas No. C11995500BT) supplemented with 10% (*v*/*v*) fetal bovine serum (FBS) (Thermo Fisher Scientific and Gibco, Waltham, MA, USA; Cas No. A5669701) at 37 °C with 5% CO_2_. The HTNV-76118 strain was propagated in Vero E6 cells as previously described [20]. Viral titers were determined using an immunofluorescence assay, and the median tissue culture infectious dose (TCID50) was calculated according to the Reed–Muench method. All virus stocks were aliquoted and stored at −80 °C.

### 2.7. Cytotoxicity Assays

The cytotoxicity of the compounds on Vero E6 cells was assessed using the MTT assay [21]. In brief, Vero E6 cells (3 × 10^4^ cells/well) were seeded in 96-well plates and treated with various concentrations of the test compounds. Negative and blank control wells were also included. The cells were incubated at 37 °C in 5% CO_2_ for 3 days. Following incubation, the cell culture medium was either replaced with phosphate-buffered saline (PBS, Servicebio^®^, Wuhan, China; Cas No. G4202-500ML) or left unchanged. Thiazolyl blue tetrazolium bromide solution (5 mg/mL) (Merck and Sigma-Aldrich, Shanghai, China; Cas No. M2128-5G) was added to each well, with further incubation for 4 h at 37 °C. The resulting supernatant of 100 µL was then removed, followed by the addition of 100 µL of 12% sodium dodecyl sulfate, dodecyl sulfate sodium salt (SDS, NeoFroxx GmbH, Einhausen, Thüringen, Germany; Cas No. 3250KG001) + 50% N, N-dimethylformamide (Macklin, Shanghai, China; Cas No. N807505-500ml). The plates were then incubated overnight at 37 °C in 5% CO_2_. The absorbance of the dissolved formazan was measured at 570/630 nm using a SpectraMax Mini microplate reader (Molecular Devices, Sunnyvale, CA, USA). The 50% cytotoxicity concentration (CC_50_) was calculated based on the absorbance data.

### 2.8. Time-of-Addition Assay (TOA)

Simultaneous: The antiviral properties of the test compounds against HTNV-76118 were comprehensively assessed in Vero E6 using quantitative reverse transcription polymerase chain reaction (qRT-PCR). In brief, Vero E6 cells (3 × 10^4^ cells/well) were seeded in 96-well plates and incubated overnight at 37 °C in 5% CO_2_. The following day, the cells were infected with the HTNV-76118 strain at a multiplicity of infection (MOI) of 1, while test compounds were simultaneously added at serially diluted concentrations. After 5 days of incubation at 37 °C in 5% CO_2_, the supernatants were harvested, and viral RNA was extracted using a DNA/RNA Extraction Kit (Prepackaged) (Vazyme, Nanjing, China; Cas No. RM401-04). The extracted RNA was then subjected to one-step qRT-PCR. The primers used for the quantitative estimation of HTNV-76118 S-segment RNA levels using real-time PCR were 5′-ACATCTGAGGAGAAGCTACGG-3′ (forward primer) and 5′-GGCAACCATGAAGAGCACAA-3′ (reverse primer).

2H pretreatment: To assess prophylactic efficacy, Vero E6 cells (3 × 10^4^ cells/well) were seeded in 96-well plates and incubated overnight at 37 °C in 5% CO_2_. The following day, the cells were treated with serially diluted concentrations of the test compounds at 37 °C for 2 h, then infected with the HTNV-76118 strain at a MOI of 1 at 37 °C for 2 h. Following viral adsorption, the cells were washed twice with PBS and maintained in fresh culture medium. After 5 days of incubation at 37 °C in 5% CO_2_, the viral RNA in the supernatants was extracted and quantified as described above.

Post entry: To determine the effect of compounds on post-entry viral replication, Vero E6 cells (3 × 10^4^ cells/well) were seeded in 96-well plates and incubated overnight at 37 °C in 5% CO_2_. The following day, the cells were infected with the HTNV-76118 strain at a MOI of 1 for 2 h. After removing the unbound virus via PBS washing, the cells were treated with different concentrations of the compounds. After 5 days of incubation at 37 °C in 5% CO_2_, the cell supernatants were collected, and viral RNA was extracted and analyzed using qRT-PCR as described above.

Virucidal: To evaluate the direct virucidal activity of the compounds, Vero E6 cells (3 × 10^4^ cells/well) were seeded in 96-well plates and incubated overnight at 37 °C in 5% CO_2_. The following day, the virus dilution solution (MOI = 1) was incubated with compounds of different concentrations at 37 °C for 2 h, then added to the cells at 37 °C for 2 h. After washing twice with PBS, the culture medium was added, and incubation at 37 °C in 5% CO_2_ was continued for 5 days. The cell supernatants were collected, and viral RNA was extracted and quantified as described above.

All experiments involving viral infections were conducted in a Biosafety Level 2 laboratory.

### 2.9. Immunofluorescence Assay

Vero E6 cells (2 × 10^5^ cells/well) were seeded in 24-well plates and incubated overnight. The following day, the culture medium was removed, and the cells were incubated at 37 °C for 2 h with 250 µL/well of diluted virus in 2% FBS DMEM, achieving a final MOI of 1. After the incubation period, 500 µL/well of the same medium was added. After 48 h, the cells were washed twice with PBS and fixed with 4% formaldehyde (Servicebio^®^, Wuhan, China; Cas No. G11101) for 15 min at room temperature (RT). The fixed cells were then washed with PBS and permeabilized with TritonX-100 (Sangon Biotech, Shanghai, China; Cas No. A600198-0500) at 0.25% in ddH_2_O for 15 min. After further washing in PBS, the cells were blocked with 5% bovine serum albumin (BSA, Sangon Biotech, Shanghai, China; Cas No. A600332-0100) for 1 h. For immunodetection, the cells were incubated overnight at 4 °C with homemade rabbit anti-HTNV nucleocapsid protein antibody (a kind gift from Prof. Manli Wang) diluted in PBS containing 1% BSA. After washing with PBS, the cells were incubated for 2 h at RT with Alexa Fluor^TM^ 488 goat anti-rabbit IgG (H+L) (Thermo Fisher Scientific and Invitrogen, Waltham, MA, USA; Cas No. A11008). The cells were washed again and mounted in 4′,6-diamidino-2-phenylindole (DAPI) (Abcam, Cambridge, UK; Cas No. ab285390) for 10 min. The samples were finally analyzed using an Axiovert 5 microscope (ZEISS, Baden-Württem, Germany). Mean gray values were analyzed using ImageJ v1.54g. All experiments involving viral infections were conducted in a Biosafety Level 2 Laboratory.

### 2.10. Molecular Docking

Molecular docking studies were accomplished using the AutoDock software package v1.5 with the hybrid Lamarckian Genetic Algorithm (LGA), as described previously [17]. The resulting complex structures of the compound and RdRp (PDB code: 8C4S, resolution: 3.27 Å) were visualized using the PyMOL Molecular Graphic System v3.1.4.

### 2.11. Statistical Analysis

Our laboratory has constructed the plasmid standard of HTNV NP (PET-28a-HTNV-NP-his tag) and established a standard curve (Figure S1). Dose–response fitting curves and bar graphs were generated using GraphPad Prism v8.0.1, and EC_50_, CC_50_, and selection index (SI) values were calculated according to the Read–Muench method. Data are represented as mean ± standard deviation (SD) unless otherwise stated. Mean gray value and statistical analysis were compared with the control group using one-way analysis of variance (ANOVA) with GraphPad Prism v8.0.1 (ns, no statistical significance; *, *p* < 0.05; **, *p* < 0.01; ***, *p* < 0.001). The calculation formula [22,23,24] is presented in the Supporting Information.

## 3. Results

### 3.1. Isolation and Structural Characterization of the Isolated Compounds

Callistemontrimer A was isolated as colorless crystals. Its molecular formula was assigned as C_40_H_54_O_9_ based on its HRESIMS ion at *m*/*z* 679.3834 [M + H]^+^ (calcd for C_40_H_55_O_9_, 679.3841) (Figure S2), indicating 14 indices of hydrogen deficiency (IHDs). The ^1^H NMR spectroscopic data (Table 1, Figure S3) revealed characteristic signals corresponding to 14 methyl groups [*δ*_H_ 1.61 × 2 (H_3_-15′/15″), 1.60 (H_3_-14″), 1.48 (H_3_-14′), 1.42 (H_3_-13′), 1.39 (H_3_-13″), 1.36 (H_3_-12″), 1.32 (H_3_-12′), 0.97 (H_3_-11), 0.94 (H_3_-10), 0.93 (H_3_-10′), 0.84 (H_3_-11′), 0.83 (H_3_-11″), and 0.68 (H_3_-9′)] and two hydroxy groups [13.31 (OH-4) and 3.42 (OH-6′)]. Based on DEPT (Figure S4) and HSQC spectra (Figure S5), the ^13^C NMR data (Table 1) displayed the presence of fourty carbon resonances, including five ketone carbonyl carbons, one aromatic ring with three oxygenated carbons [*δ*_C_ 160.6 (C-4), 152.6 (C-6), 151.1 (C-2), 109.8 (C-3), 109.3 (C-5), and 107.1 (C-1)], one hemiketal carbon [*δ*_C_ 211.8 (C-4″)], one double bond [*δ*_C_ 166.4 (C-6″) and 113.5 (C-1″)], four quaternary carbons [*δ*_C_ 57.8 (C-3′), 56.3 (C-3″), 54.9 (C-5′), and 47.2 (C-5″)], six methines [*δ*_C_ 45.8 (C-1′), 32.1 (C-8′), 29.0 (C-7′), 25.7 (C-7″), 25.2 (C-9″), and 24.9 (C-9)], two methylenes [*δ*_C_ 53.8 (C-8) and 56.3 (C-3″)], and fourteen methyls [*δ*_C_ 26.9 (CH_3_-13′), 25.2 (CH_3_-15″), 25.1 × 2 (CH_3_-15′/14″), 24.7 (CH_3_-13″), 24.4 (CH_3_-12″), 23.8 × 2 (CH_3_-11′/11″), 22.8 (CH_3_-11), 22.6 (CH_3_-10), 22.1 (CH_3_-12′), 20.2 (CH_3_-10′), 19.5 (CH_3_-14′), and 15.9 (CH_3_-9′)]. These functionalities accounted for nine of the fourteen IHDs, indicating that callistemontrimer A was pentacyclic.

**Figure 2:**
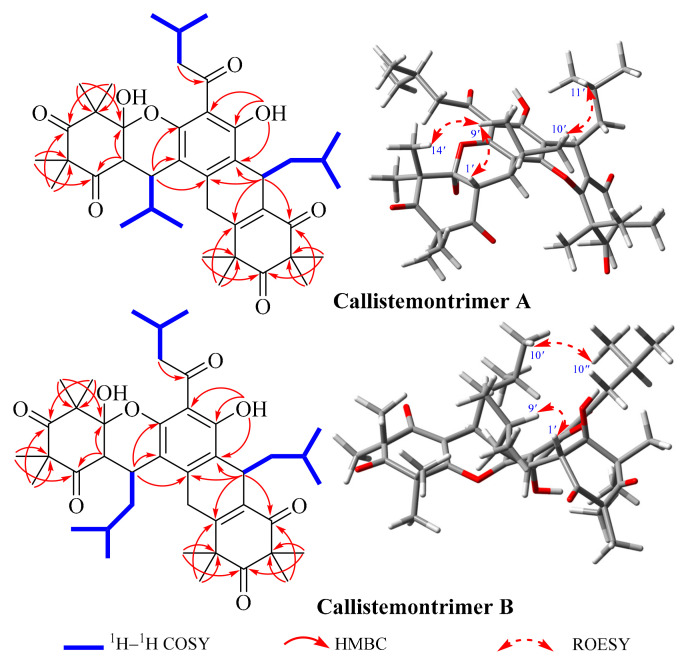
Key ^1^H–^1^H COSY, HMBC, and ROESY correlations of callistemontrimer A and B.

**Figure 3:**
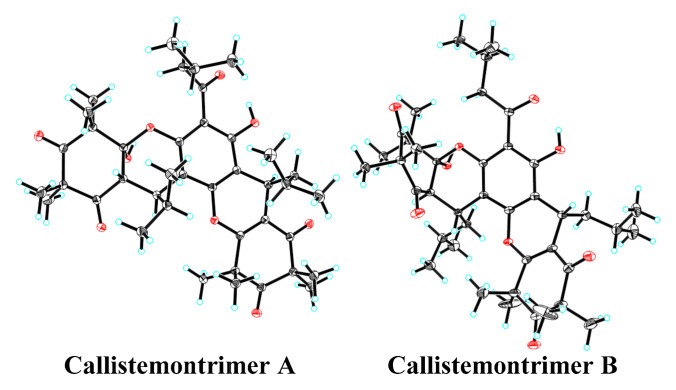
X-ray crystal structures of callistemontrimer A and B.

The planar structure and configuration of callistemontrimer A were determined through analysis of its ^1^H–^1^H correlation spectroscopy (COSY) (Figure S6), ^1^H detected heteronuclear multiple bond correlation (HMBC) (Figure S7), and rotating frame Overhauser effect spectroscopy (ROESY) spectra, along with ECD calculations and X-ray diffraction data. The ^1^H–^1^H COSY spectrum revealed three partial fragments of H_2_-8–H-9–H_3_-10/H_3_-11, H-1′–H-7′–H-8′–H_3_-9′/H_3_-10′, and H-7″–H_2_-8″–H-9″–H_3_-10″/H_3_-11″. Key HMBC correlations included H_2_-8 to C-7, OH-6 to C-3/C-4/C-5, H-1′ to C-2′/C-6′, H-7′ to C-1/C-2/C-6, H_3_-11′/H_3_-12′ to C-2′/C-3′/C-4′, H_3_-13′/H_3_-14′ to C-4′/C-5′/C-6′, H-7″ to C-5/C-6/C-1″/C-2″/C-6″, H_3_-12″/H_3_-13″ to C-2″/C-3″/C-4″, and H_3_-14″/H_3_-15″ to C-4″/C-5″/C-6″, indicating that two *β*-triketone units were attached to the phloroglucinol moiety via two pyran rings. The ROESY correlations (Figure 2 and Figure S8) between H-1′ and H_3_-9′ and between H_3_-10′ and H_3_-11″ indicated that these protons were cofacial. However, the relative configuration of OH-6′ could not be resolved with the available data. Fortunately, X-ray diffraction analysis (Figure 3) of callistemontrimer A not only confirmed the proposed structure but also revealed that the configuration of OH-6′ was opposite to that of H-1′. Subsequent chiral separation and ECD calculations (Figure S9) established the absolute configurations of (+)-callistemontrimer A and (–)-callistemontrimer A to be (1′*R*,6′*S*,7′*S*,7″*R*) and (1′*S*,6′*R*,7′*R*,7″*S*), respectively.

Callistemontrimer B was assigned the molecular formula C_41_H_56_O_9_, as determined based on its HRESIMS ion at *m*/*z* 693.4000 [M + H]^+^ (calcd for C_41_H_57_O_9_, 693.3997) (Figure S10). A comparison of the NMR data of callistemontrimer B with those of callistemontrimer A (Table 2 and Figures S11–S13) suggested a similar planar structure, with the primary difference being the substitution of the C-1′ isobutyl group with an isopentyl group. This substitution was confirmed based on key HMBC correlations from H-1′ to C-2′/C-6′, as well as the key fragment of H-1′–H-7′–H_2_-8′–H-9′–H_3_-10′/H_3_-11′, as evidenced by its ^1^H–^1^H COSY spectrum (Figures S14 and S15). In addition, the ROESY correlations of H-1′ with H_3_-10′ and H_3_-10′ with H_3_-10″ indicated that these protons were positioned on the same side (Figure S16). However, X-ray diffraction data (Figure 3) confirmed that callistemontrimer B was also a racemic mixture, with the same configuration for OH-6′ and H-1′. Further ECD calculations established the absolute configurations of (+)-callistemontrimer B and (–)-callistemontrimer B as (1′*S*,6′*S*,7′*R*,7″*S*) and (1′*R*,6′*R*,7′*S*,7″*R*), respectively (Figure S17).

In addition, two previously reported phloroglucinol dimers, rhodomyrtosone B [25] and rhodomyrtone [26], were identified by comparing their NMR and MS spectra with those reported in the literature (Figure 1). Notably, this represents the first instance of these known dimers being isolated from a *Callistemon* species.

### 3.2. Cytotoxicity and Antiviral Activities of Isolated Compounds

**Figure 4:**
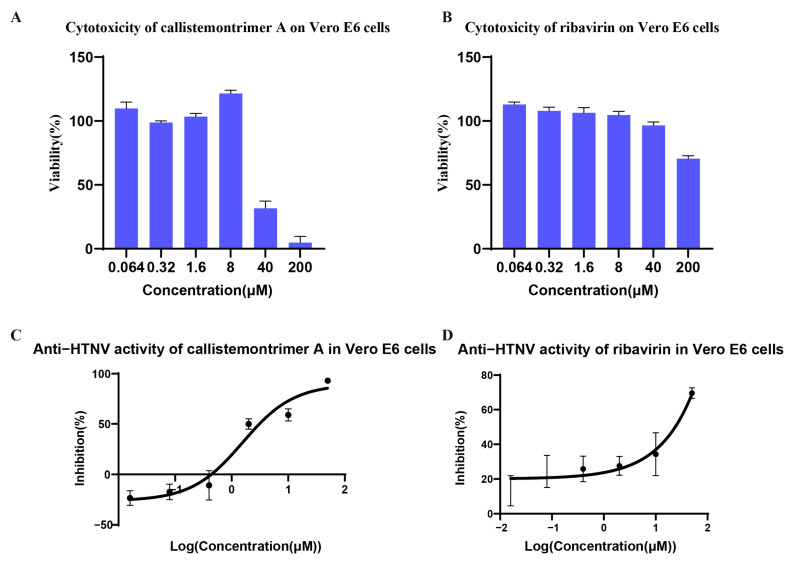
Antiviral activity of callistemontrimer A and ribavirin. The cytotoxicity of callistemontrimer A and ribavirin on Vero E6 (**A**,**B**) was determined using the MTT assay after co-incubation of the compounds with the cells for 3 days. Dose–response curves show quantitation of HTNV S segment RNA levels in Vero E6 cells (**C**,**D**) after HTNV treatment with gradient-diluted compounds for 5 days. All data represent means ± standard deviation (SD) for two independent replicate experiments.

The anti-HTNV activities of all isolated compounds were initially assessed using Vero E6 cells [27], which were subsequently used for further mechanistic investigations. Ribavirin served as a positive control in all assays. The in vitro anti-HTNV activities of the four phloroglucinol oligomers isolated from the leaves of *C. rigidus*, including callistemontrimer A, callistemontrimer B, rhodomyrtosone B, and rhodomyrtone, were evaluated. As shown in Table 3 and Figure 4 and Figure S18, callistemontrimer A, a newly identified phloroglucinol trimer, exhibited the highest SI value of 7.28, prompting its selection for detailed follow-up studies. Notably, all four compounds demonstrated stronger anti-HTNV potency than the positive control, ribavirin.

**Figure 5:**
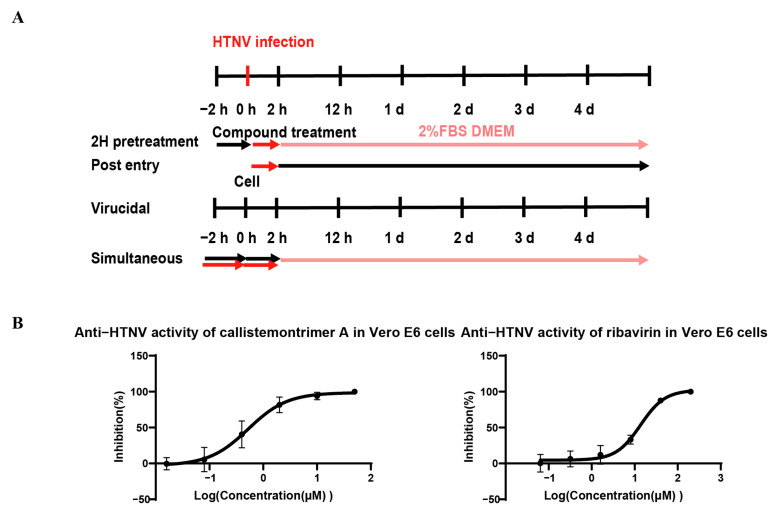
The time-of-addition assay of callistemontrimer A and ribavirin. (**A**) Various treatment regimens were applied to HTNV-infected Vero E6 cells at a MOI of 1. 2H pretreatment: compound treatment for 2 h pre-infection; post entry: compound treatment for 2 h post-infection; virucidal: compounds incubated with virus for 2 h, followed by cell treatment for 2 h. (**B**) The inhibitory effect of callistemontrimer A and ribavirin on HTNV replication. Dose–response curves show quantitation of HTNV S segment RNA levels in Vero E6 cells after HTNV treatment with gradient-diluted compounds for 5 days. All data represent means ± standard deviation (SD) for two independent replicate experiments.

To elucidate the antiviral mechanism of callistemontrimer A, further experiments using different drug–virus treatments were conducted (Figure 5A). The results indicated that both callistemontrimer A and ribavirin exhibited inhibitory effects on viral replication in Vero E6 cells, with EC_50_ values of 0.65 ± 0.45 and 13.16 ± 0.40 μM, respectively (Table 4 and Figure 5B). However, neither compound demonstrated protective effects on HTNV-infected Vero E6 cells, with EC_50_ values exceeding 50 μM and 200 μM, respectively (Table 4). Furthermore, neither compound exhibited a direct virucidal effect on HTNV (Table 4). These findings suggest that callistemontrimer A is more likely to exert its antiviral properties by targeting the viral replication stage.

To further visualize the inhibitory effects of callistemontrimer A and ribavirin on viral replication in HTNV-infected Vero E6 cells, immunofluorescence experiments were conducted to detect viral core proteins after treatment with varying concentrations of the compounds. The results indicated that callistemontrimer A effectively suppressed viral replication at concentrations of both 10 μM and 20 μM (Figure S19). Notably, at equivalent concentrations, callistemontrimer A demonstrated superior inhibitory effects compared to ribavirin. However, quantitative analysis of mean gray values using ImageJ revealed no significant differences among the treatment groups.

### 3.3. Molecular Docking

**Figure 6:**
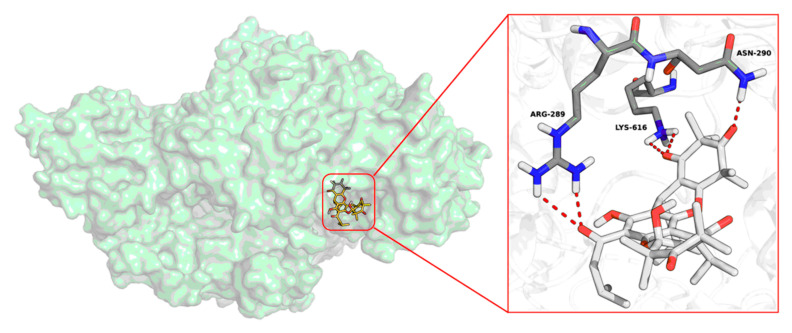
Molecular docking results of callistemontrimer A with RdRp (PDB code: 8C4S).

The RdRp of HV, with a molecular weight ranging from 250 to 280 kDa, consists of three subdomains: the thumb, finger, and palm. These subdomains facilitate viral mRNA synthesis and genome replication within the cytoplasm. In addition to its transcriptase and replicase activities, RdRp also functions as an RNA helicase but lacks the enzymatic activity required for capping and proofreading, making it a crucial target for the development of anti-HV drugs [28,29]. Our experimental results demonstrated that the antiviral effects of callistemontrimer A may disrupt the viral replication phase. Given the pivotal role of RdRp in HV genome replication and transcription [30], molecular docking was conducted to elucidate the chemical interactions between callistemontrimer A and RdRp (PDB code:8C4S). The results indicated that callistemontrimer A formed five hydrogen bonds with amino acids ARG-289, LYS-616, and ASN-290 within the bioactive pocket (Figure 6). These observations suggest that callistemontrimer A may be a promising candidate for anti-HV therapy.

## 4. Discussion

HV infections pose a significant global health threat. Prompt and efficient antiviral therapy can markedly improve the prognosis of a variety of viral diseases [31]. Consequently, the development of effective, low-toxicity anti-HV drugs for clinical use is urgently required. Despite recent advancements in the development of therapeutic drugs and preventive measures against HV infection, significant challenges remain due to the lack of appropriate animal models and an incomplete understanding of the molecular mechanisms underlying HV infection. Natural products and their structural analogs are well-known for their extensive biological activity and diverse chemical structures, making them a major source of traditional oral medications, with important contributions to cancer and infectious disease treatment [10]. Recent studies have highlighted the potential of various natural product classes, including alkaloids [32], coumarins [33], flavonoids [34], and algal lectins [35], as promising anti-HV agents. However, the anti-HV potential of other plant-derived natural products remains largely unexplored, warranting further investigation.

Four phloroglucinol oligomers (callistemontrimer A, callistemontrimer B, rhodomyrtosone B, and rhodomyrtone) were isolated from the leaves of *C. rigidus* and structurally characterized. The anti-HTNV activities of these compounds were evaluated, marking the first identification of oligomeric phloroglucinols with potential anti-HTNV properties. Notably, all four isolates displayed stronger anti-HTNV activity than the positive control ribavirin, highlighting their potential as lead compounds for antiviral drug development. To enhance efficacy, structural optimization—such as incorporating hemiketal hydroxyl groups or additional phloroglucinol units and other functional moieties—may represent a promising strategy for advancing these candidates toward potent anti-HTNV therapeutics.

Among the four isolated compounds, callistemontrimer A was selected for further investigation due to its high SI value. Ribavirin, a broad-spectrum antiviral drug, primarily targets the genome of RNA viruses, inosine monophosphate dehydrogenase, inosine 5′-monophosphate dehydrogenase, and other sites. In this study, ribavirin served as a positive control for comparative analysis of the antiviral mechanism of callistemontrimer A through different drug treatments. Preliminary findings suggested that, similar to ribavirin, callistemontrimer A targeted the viral replication stage. Notably, at equivalent concentrations, callistemontrimer A demonstrated superior inhibitory effects compared to ribavirin. Molecular docking analysis revealed interactions between callistemontrimer A and RdRp of HTNV, although the precise molecular mechanisms remain to be fully elucidated. Thus, callistemontrimer A shows promise as a new lead compound for anti-HV drug research, potentially advancing the development of novel therapeutic agents.

## 5. Conclusions

In conclusion, this phytochemical investigation of *C. rigidus* leaves collected from a temperate region in China resulted in the isolation of four phloroglucinol oligomers, including two newly identified phloroglucinol trimers (callistemontrimer A and B) and two known phloroglucinol dimers (rhodomyrtosone B and rhodomyrtone). Importantly, all compounds demonstrated significant antiviral activity against HTNV, with callistemontrimer A exhibiting exceptional potency in inhibiting viral replication, likely through interaction with HTNV RdRp. Molecular docking studies provided further insights into the predicted binding mode and potential mechanism of action. These findings not only offer promising natural compounds for the development of novel therapeutic agents against HV-related diseases but also provide valuable evidence to support further structural optimization and pharmacological evaluation.

## Figures and Tables

**Table 1:** ^13^C (150 MHz) and ^1^H (600 MHz) NMR spectroscopic data of callistemontrimer A in CDCl_3_.

No.	*δ*_C_	*δ*_H_ (Mult, *J* in Hz)	No.	*δ*_C_	*δ*_H_ (Mult, *J* in Hz)	No.	*δ*_C_	*δ*_H_ (Mult, *J* in Hz)
1	107.1		1′	45.8	3.66 (d, 5.9)	1″	113.5	
2	151.1		2′	205.1		2″	197.6	
3	109.8		3′	57.8		3″	56.3	
4	160.6		4′	214.0		4″	211.8	
5	109.3		5′	54.9		5″	47.2	
6	152.6		6′	100.2		6″	166.4	
7	205.6		7′	29.0	4.17 (brdd, 5.9, 3.9)	7″	25.7	4.19 (t, 6.5)
8	53.8	3.17 (dd, 16.9, 6.9)	8′	32.1	2.35 (m)	8″	44.3	1.58 (ddd, 18.5, 13.2, 6.5)
		2.80 (dd, 16.6, 6.9)						1.41 (overlapped)
9	24.9	2.26 (brheptet, 6.7)	9′	15.9	0.68 (d, 6.9)	9″	25.2	1.40 (overlapped)
10	22.6	0.94 (d, 6.7)	10′	20.2	0.93 (d, 6.9)	10″	23.4	0.69 (d, 6.4)
11	22.8	0.97 (d, 6.7)	11′	23.8	0.84 (d, 6.3)	11″	23.8	0.83 (d, 6.4)
OH-4		13.31 (s)	12′	22.1	1.32 (s)	12″	24.4	1.36 (s)
OH-6′		3.42 (s)	13′	26.9	1.42 (s)	13″	24.7	1.39 (s)
			14′	19.5	1.48 (s)	14″	25.1	1.60 (s)
			15′	25.1	1.61 (s)	15″	25.2	1.61 (s)

**Table 2:** ^13^C (150 MHz) and ^1^H (600 MHz) NMR spectroscopic data of callistemontrimer B in CDCl_3_.

No.	*δ*_C_	*δ*_H_ (Mult., *J* in Hz)	No.	*δ*_C_	*δ*_H_ (Mult., *J* in Hz)	No.	*δ*_C_	*δ*_H_ (Mult., *J* in Hz)
1	104.3		1′	45.8	3.33 (brs)	1″	114.6	
2	150.6		2′	210.8		2″	197.3	
3	108.5		3′	54.8		3″	56.2	
4	161.4		4′	212.1		4″	210.6	
5	109.2		5′	54.3		5″	47.2	
6	153.5		6′	100.6		6″	166.7	
7	205.4		7′	30.2	3.86 (brdd, 12.3, 4.5)	7″	25.0	4.27 (t, 6.0)
8	54.0	2.80 (dd, 18.2, 6.3)	8′	41.8	2.19 (td, 12.3, 3.2)	8″	46.2	1.38 (m)
		2.66 (dd, 18.2, 7.1)			1.73 (ddd, 13.5, 11.1, 4.5)			
9	23.9	2.27 (brheptet, 6.7)	9′	25.5	1.87 (m)	9″	25.2	1.44 (m)
10	22.4	0.89 (d, 6.7)	10′	20.8	1.16 (d, 6.4)	10″	23.3	0.84 (d, 6.3)
11	22.6	0.98 (d, 6.7)	11′	24.0	1.00 (d, 6.5)	11″	23.0	0.86 (d, 6.3)
OH-4		13.80 (s)	12′	24.6	0.91 (s)	12″	24.2	1.34 (s)
			13′	26.4	1.34 (s)	13″	24.4	1.39 (s)
			14′	22.1	1.37 (s)	14″	24.7	1.51 (s)
			15′	15.8	1.54 (s)	15″	24.9	1.62 (s)

**Table 3:** Anti-HTNV activities of compounds on Vero E6 cell lines.

Name of Compounds	EC_50_ (μM)	*p* Value	CC_50_ (μM)	SI
Callistemontrimer A	4.41 ± 3.40	ns	32.09 ± 6.00	7.28
Callistemontrimer B	2.79 ± 1.40	ns	16.89 ± 1.00	6.05
Rhodomyrtosone B	2.69 ± 0.32	ns	7.44 ± 1.15	2.77
Rhodomyrtone	0.30 ± 0.03	*	5.11 ± 2.49	17.03
Ribavirin	20.54 ± 1.63	-	>200	>9.74

Note: The cytotoxicity of callistemtrier A and ribavirin towards Vero E6 cells was determined using the MTT assay after co-incubation of the compounds with the cells for 3 days, and the CC_50_ value was calculated. The antiviral activity of the compounds was measured after HTNV treatment with gradient-diluted compounds for 5 days, and the EC_50_ value was calculated. Among of them, ns, no statistical significance; *, *p* < 0.05.

**Table 4:** Time-of-addition assay of callistemontrimer A and ribavirin.

Time-of-Addition Assay	EC_50_(μM)	
	Callistemontrimer A	Ribavirin
2H pretreatment	>50	>200
Post entry	0.65 ± 0.45	13.16 ± 0.40
Virucidal	>50	>200

## Data Availability

The datasets generated and analyzed in this study are available from the corresponding author upon reasonable request.

## Supplementary Materials

The following supporting information can be downloaded at: https://www.mdpi.com/article/10.3390/v17070916/s1, Calculation formula; Figure S1: The standard curve constructed based on the HTNV NP plasmid standard; Figure S2: HRESIMS spectrum of callistemontrimer A; Figure S3: ^1^H NMR spectrum (600 MHz, CDCl_l3_) of callistemontrimer A; Figure S4: ^13^C NMR spectrum (150 MHz, CDCl_l3_) of callistemontrimer A; Figure S5: HSQC spectrum of callistemontrimer A; Figure S6: ^1^H–^1^H COSY spectrum of callistemontrimer A; Figure S7: HMBC spectrum of callistemontrimer A; Figure S8: ROESY spectrum of callistemontrimer A; Figure S9: Calculated ECD data of callistemontrimer A; Figure S10: HRESIMS spectrum of callistemontrimer B; Figure S11: ^1^H NMR spectrum (600 MHz, CDCl_l3_) of callistemontrimer B; Figure S12: ^13^C NMR spectrum (150 MHz, CDCl_l3_) of callistemontrimer B; Figure S13: HSQC spectrum of callistemontrimer B; Figure S14: ^1^H–^1^H COSY spectrum of callistemontrimer B; Figure S15: HMBC spectrum of callistemontrimer B; Figure S16: ROESY spectrum of callistemontrimer B; Figure S17: Calculated ECD data of callistemontrimer B; Figure S18: Anti-HTNV activities of compounds; Figure S19: Visualization of the inhibitory effects of callistemontrimer A and ribavirin on HTNV replication.
