# Supplementary material for: Phloroglucinol Oligomers from Callistemon rigidus as Novel Anti-Hantavirus Replication Agents
Source: Viruses. 2025 Jun 27;17(7):916. doi: 10.3390/v17070916 (PMC12299576; doi:10.3390/v17070916)
Supplement: Supplementary file 1 [file viruses-17-00916-s001.zip › viruses-3634307-supplementary.pdf]

Supplementary information

Phloroglucinol oligomers from *Callistemon rigidus* as novel anti-hantavirus replication agents

Jin-Xuan Yang[1,3,†], E-E Luo[2,3,†], Yue-Chun Wu[1,3], Kai Zhao[1,3], Wei Hou[4,*], Mu-Yuan Yu[2,*], Xu-Jie Qin[2,3,*], Xing-Lou Yang[1,3,*]

[1] Key Laboratory of Genetic Evolution & Animal Models, Yunnan International Joint Laboratory of Zoonotic Viruses, Yunnan Key Laboratory of Biodiversity Information, Kunming Institute of Zoology, Chinese Academy of Sciences, Kunming 650201, China yangjinxuan260@163.com (J.Y.); wuyuechun@mail.kiz.ac.cn (Y.W.); zhaokai@mail.kiz.ac.cn (K.Z.)

[2] State Key Laboratory of Phytochemistry and Natural Medicines, Kunming Institute of Botany, Chinese Academy of Sciences, Kunming 650201, China luoee@mail.kib.ac.cn (E.L.)

[3] University of Chinese Academy of Sciences, Beijing 100049, China

[4] State Key Laboratory of Virology, Institute of Medical Virology, School of Basic Medical Sciences, Wuhan University, Wuhan 430071, Hubei, China

[*] Correspondence: houwei@whu.edu.cn (W.H.); yumuyuan@mail.kib.ac.cn (M.Y.); qinxujie@mail.kib.ac.cn (X.Q.); yangxinglou@mail.kiz.ac.cn (X.Y.),

[†] These authors contributed equally to this work.

Supplementary information contents

Calculation formula 1

Fig. S1 The standard curve constructed based on the HTNV NP plasmid standard 1

Fig. S2 HRESIMS spectrum of callistemontrimer A 2

Fig. S3 ^{1}H NMR spectrum (600 MHz, $CDCl_3$) of callistemontrimer A 3

Fig. S4 ^{13}C NMR spectrum (150 MHz, $CDCl_3$) of callistemontrimer A 3

Fig. S5 HSQC spectrum of callistemontrimer A 4

Fig. S6 ^{1}H–^{1}H COSY spectrum of callistemontrimer A 4

Fig. S7 HMBC spectrum of callistemontrimer A 5

Fig. S8 ROESY spectrum of callistemontrimer A 5

Fig. S9 Calculated ECD data of callistemontrimer A 6

Fig.S10 HRESIMS spectrum of callistemontrimer B 6

Fig. S11 ^{1}H NMR spectrum (600 MHz, $CDCl_3$) of callistemontrimer B 7

Fig. S12 ^{13}C NMR spectrum (150 MHz, $CDCl_3$) of callistemontrimer B 7

Fig. S13 HSQC spectrum of callistemontrimer B 8

Fig. S14 ^{1}H–^{1}H COSY spectrum of callistemontrimer B 8

Fig. S15 HMBC spectrum of callistemontrimer B 9

Fig. S16 ROESY spectrum of callistemontrimer B 9

Fig. S17 Calculated ECD data of callistemontrimer B 10

Fig. S18 Anti-HTNV activities of compounds 10

Fig. S19 Visualization of the inhibitory effects of callistemontrimer A and ribavirin on HTNV replication 11

Calculation formulas

$$\text{Viability}=\left(\frac{\text{OD value of the experimental well}-\text{OD value of the blank well}}{\text{OD value of the negative well}-\text{OD value of the blank well}}\right)\times 100\%$$

$$\text{Inhibition}=\left(1-\frac{\text{relative virus nucleic acid}/\mu\text{L of experimental well}}{\text{relative virus nucleic acidr}/\mu\text{L of positive well}}\right)\times 100\%$$

$$CC_{50}=10EXP[\lg(>50\%\ \text{concentration value})+\left(\frac{>50\%\ \text{percentage viability}-50}{(>50\%\ \text{percentage viability})-(<50\%\ \text{percentage viability})}\right)\times \lg(\text{dilution ratio})]$$

$$EC_{50}=10EXP[\lg(>50\%\ \text{concentration value})-\left(\frac{>50\%\ \text{percentage inhibition}-50}{(>50\%\ \text{percentage inhibition})-(<50\%\ \text{percentage inhibition})}\right)\times \lg(\text{dilution ratio})]$$

$$SI=\frac{CC_{50}}{EC_{50}}$$

Fig. S1 The standard curve constructed based on the HTNV NP plasmid standard.

Our laboratory has constructed the plasmid standard of HTNV NP (PET-28a-HTNV-NP-his tag) and established a standard curve, which can convert CT values into relative virus nucleic acid. The standard curve is plotted with the logarithm of the concentration of the plasmid standard (log10) as the X-axis and the Ct value as the Y-axis.

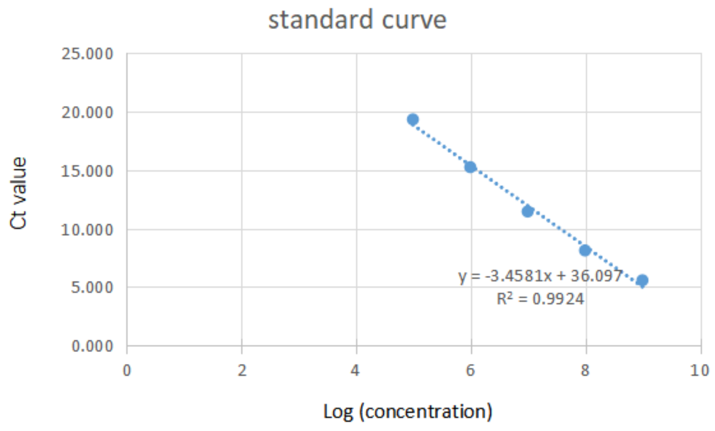

Fig. S2 HRESIMS spectrum of callistemontrimer A.

User Spectra

Fragmentor Voltage	Collision Energy	Ionization Mode
135	0	ESI

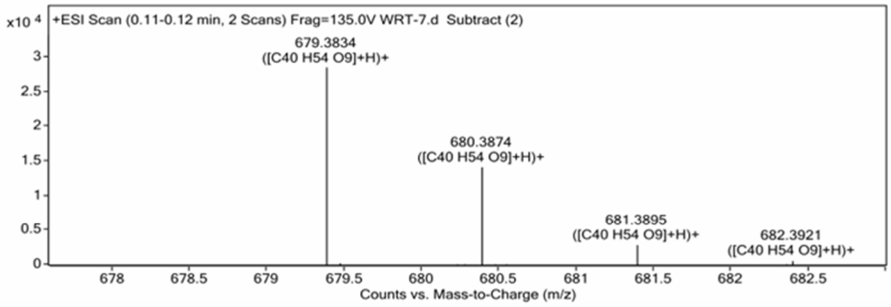

Peak List

m/z	z	Abund	Formula	Ion
74.0967	1	57330.64		
88.1122	1	15584.58		
102.1277	1	25136.11		
661.3728	1	10167.07		
679.3834	1	28563.01	C40 H54 O9	(M+H)+
680.3874	1	14232.2	C40 H54 O9	(M+H)+
1379.7413	1	24912.88		
1380.7444	1	21774.4		
1393.7554	1	16500.78		
1394.7596	1	13447.48		

Formula Calculator Element Limits

Element	Min	Max
C	3	60
H	0	120
O	0	20

Formula Calculator Results

Formula	CalculatedMass	CalculatedMz	Mz	Diff. (mDa)	Diff. (ppm)	DBE
C40 H54 O9	678.3768	679.3841	679.3834	0.70	1.03	14.0000

Fig. S3 ^{1}H NMR spectrum (600 MHz, $CDCl_3$) of callistemontrimer A.

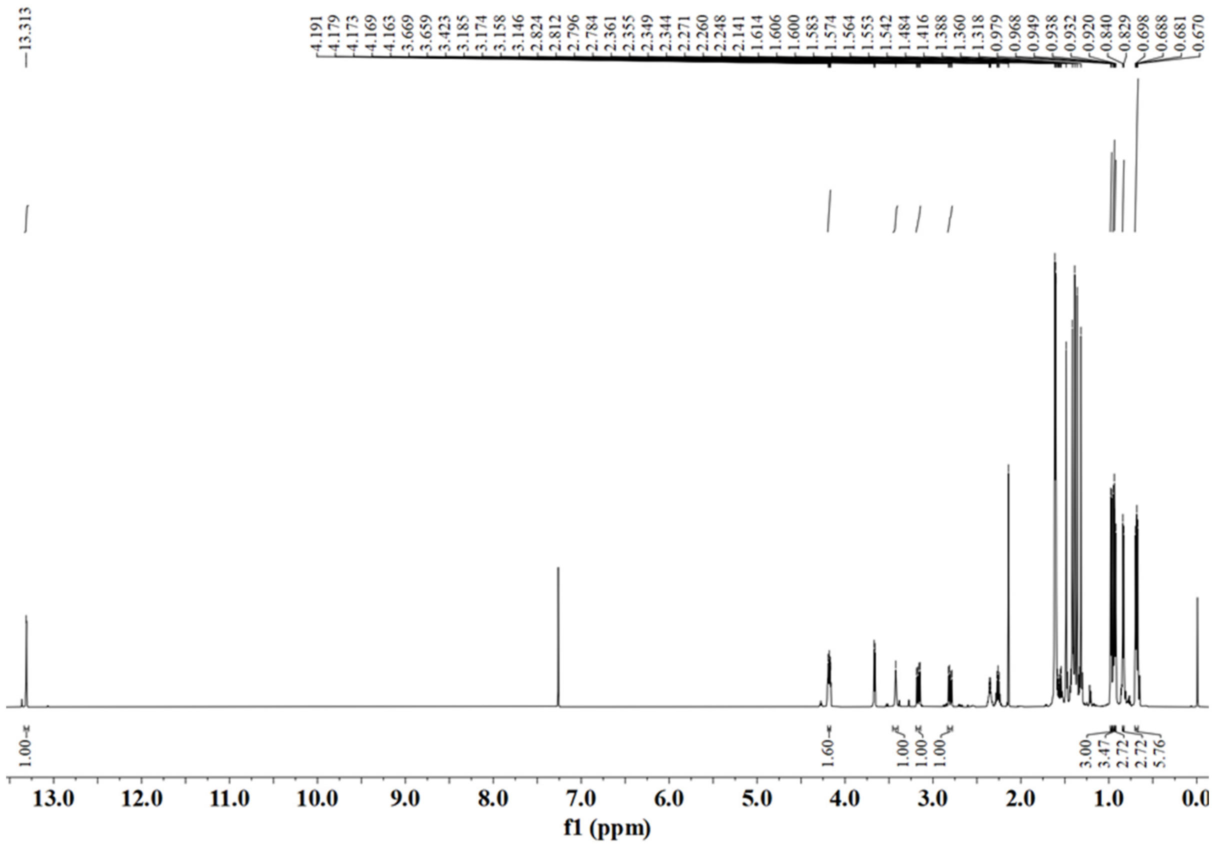

Fig. S4 ^{13}C NMR spectrum (150 MHz, $CDCl_3$) of callistemontrimer A.

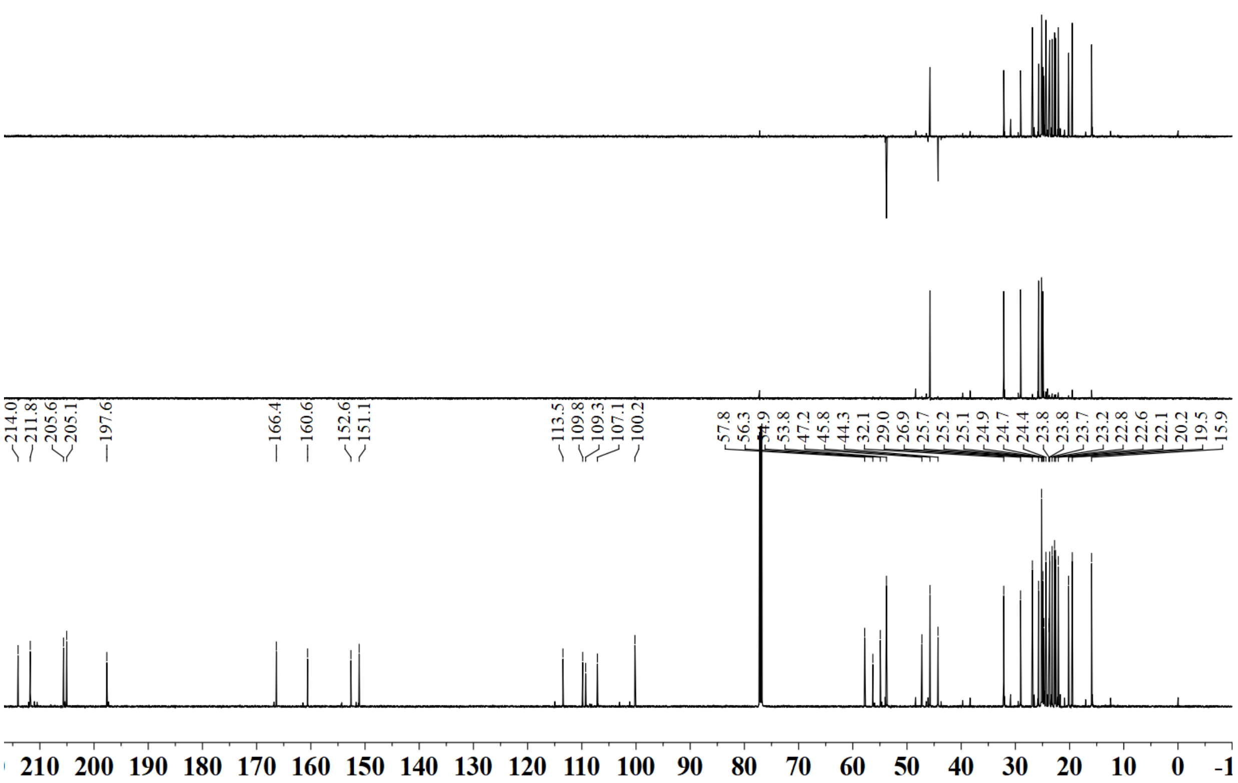

Fig. S5 HSQC spectrum of callistemontrimer A.

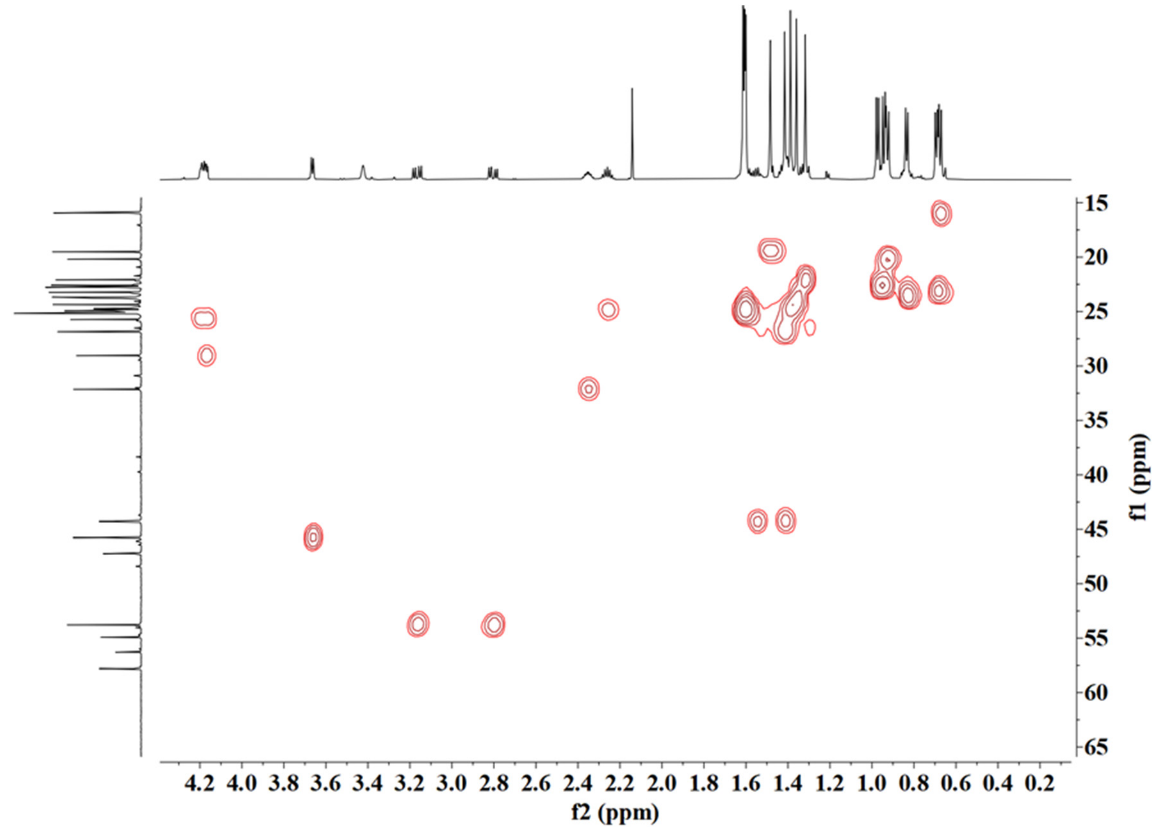

Fig. S6 ^{1}H–^{1}H COSY spectrum of callistemontrimer A.

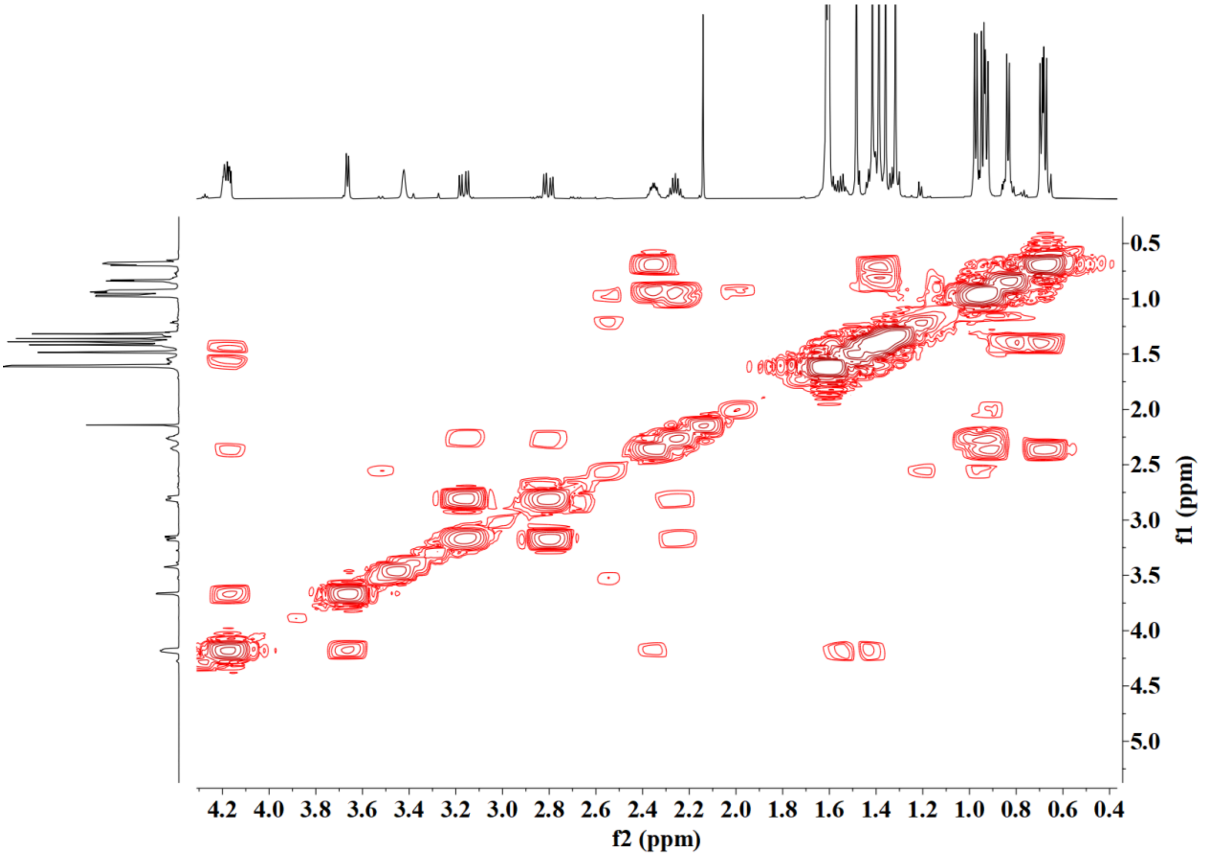

Fig. S7 HMBC spectrum of callistemontrimer A.

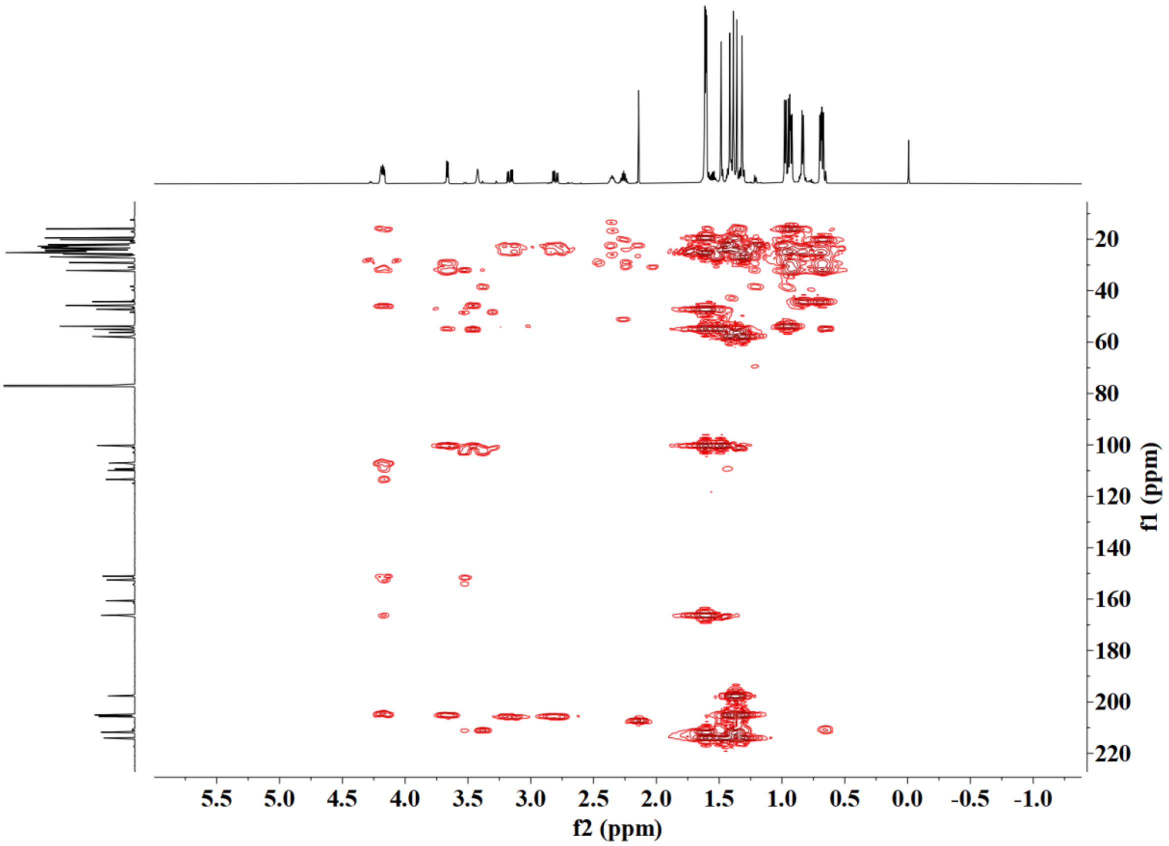

Fig. S8 ROESY spectrum of callistemontrimer A.

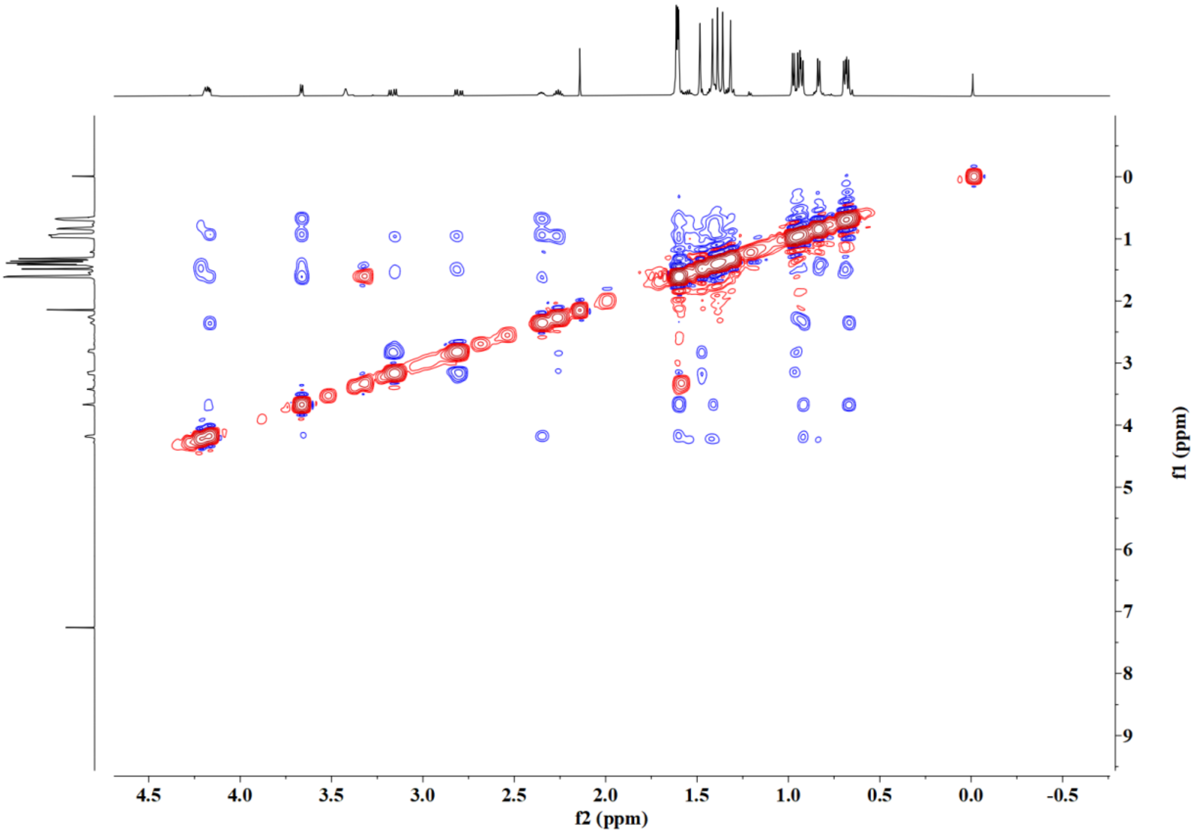

Fig. S9 Calculated ECD data of callistemontrimer A.

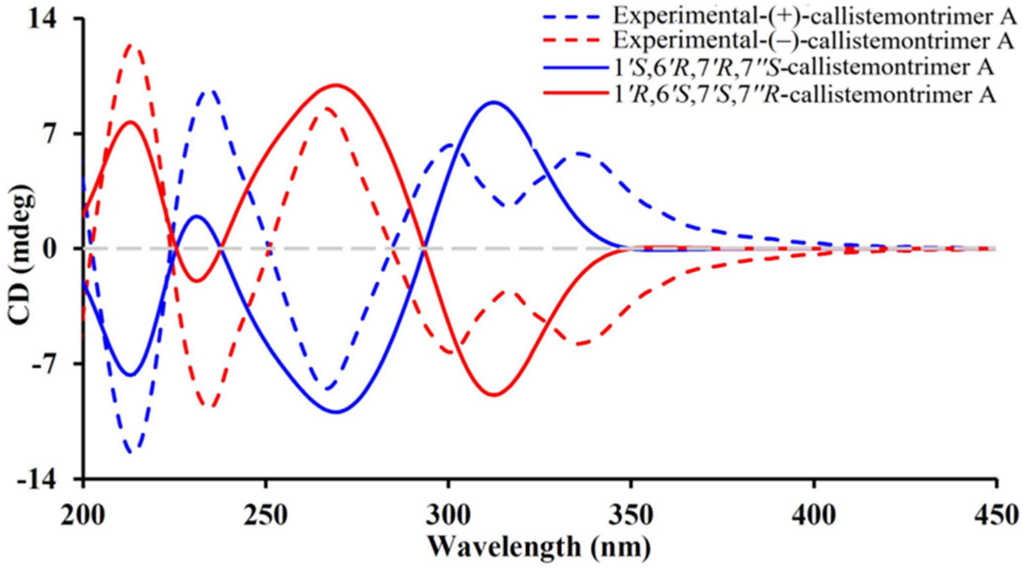

Fig.S10 HRESIMS spectrum of callistemontrimer B.

User Spectra

Fragmentor Voltage	Collision Energy	Ionization Mode
200	0	ESI

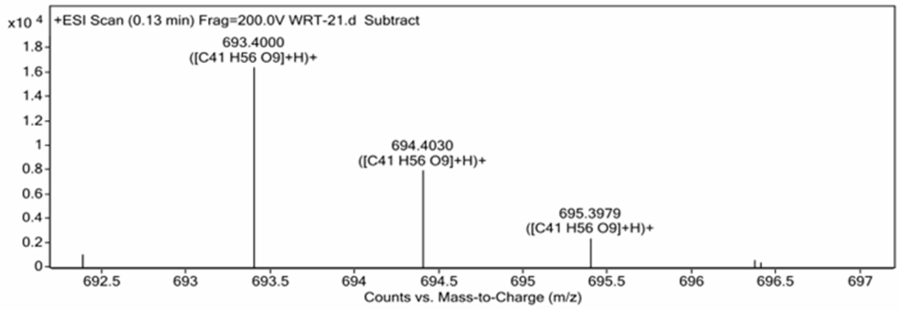

Peak List

m/z	z	Abund	Formula	Ion
429.2277	1	130917.67		
430.2309	1	35226.79		
443.2433	1	35618.53		
451.2091	1	64452.68		
452.213	1	16260.82		
502.3171	1	11212.83		
693.4	1	16406.8	C41 H56 O9	(M+H)+
879.4303	1	97772.14		
880.433	1	48950.81		
881.4368	1	16428.58		

Formula Calculator Element Limits

Element	Min	Max
C	3	60
H	0	120
O	0	20

Formula Calculator Results

Formula	CalculatedMass	CalculatedMz	Mz	Diff. (mDa)	Diff. (ppm)	DBE
C41 H56 O9	692.3924	693.3997	693.4000	-0.30	-0.43	14.0000

Fig. S11 ^{1}H NMR spectrum (600 MHz, $CDCl_3$) of callistemontrimer B.

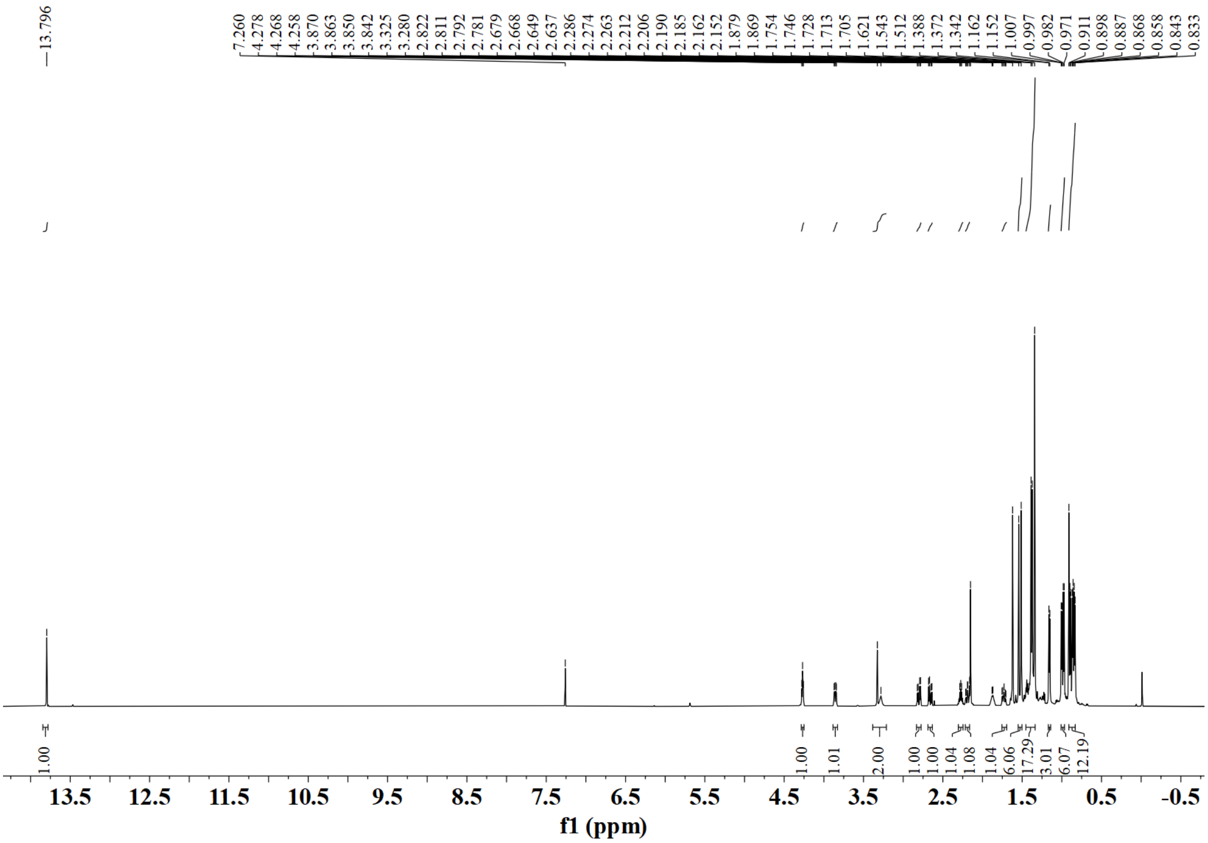

Fig. S12 ^{13}C NMR spectrum (150 MHz, $CDCl_3$) of callistemontrimer B.

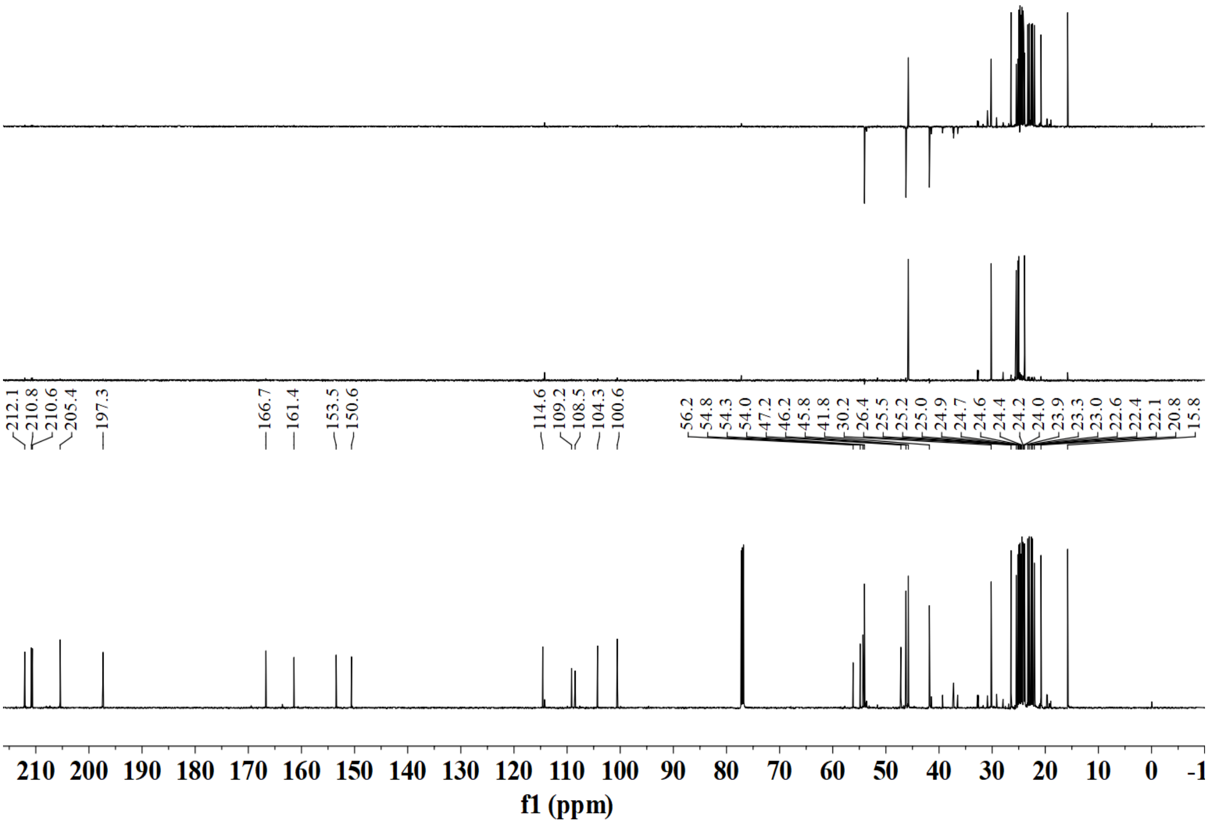

Fig. S13 HSQC spectrum of callistemontrimer B.

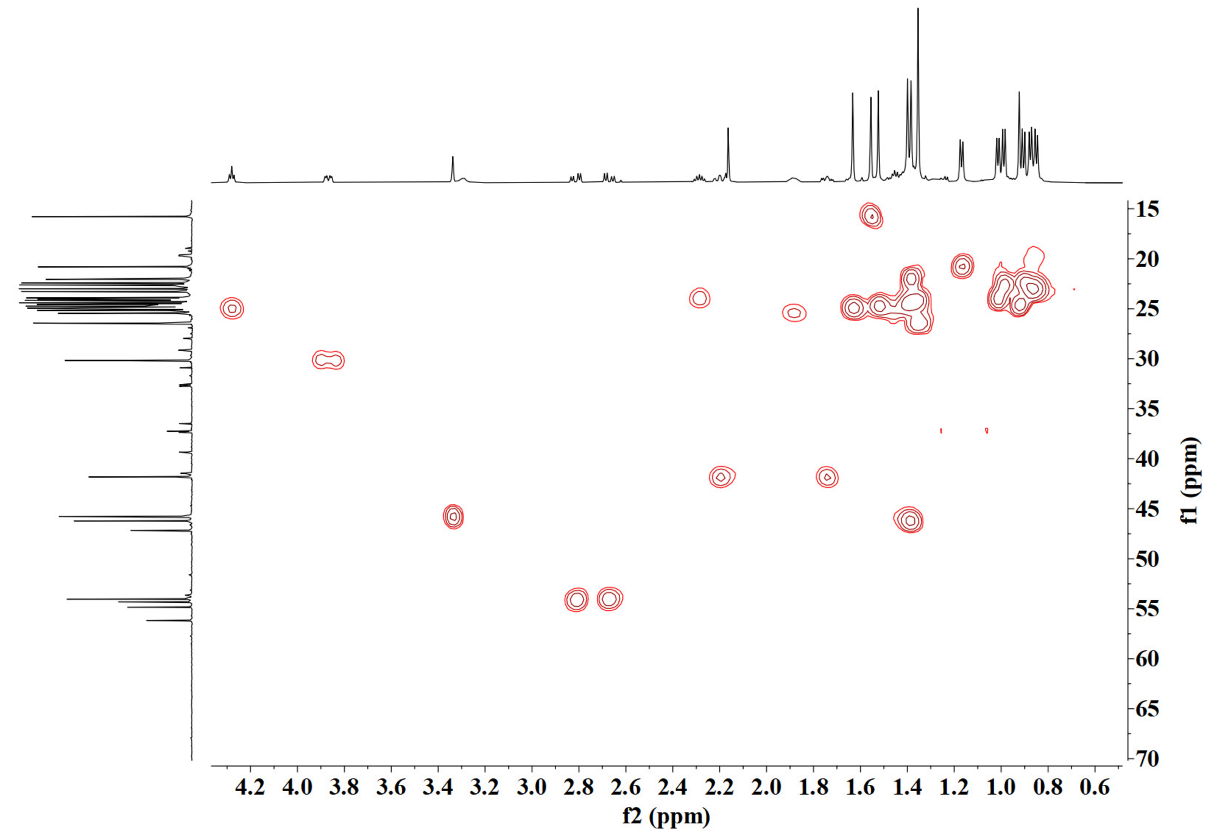

Fig. S14 ^1H–^1H COSY spectrum of callistemontrimer B.

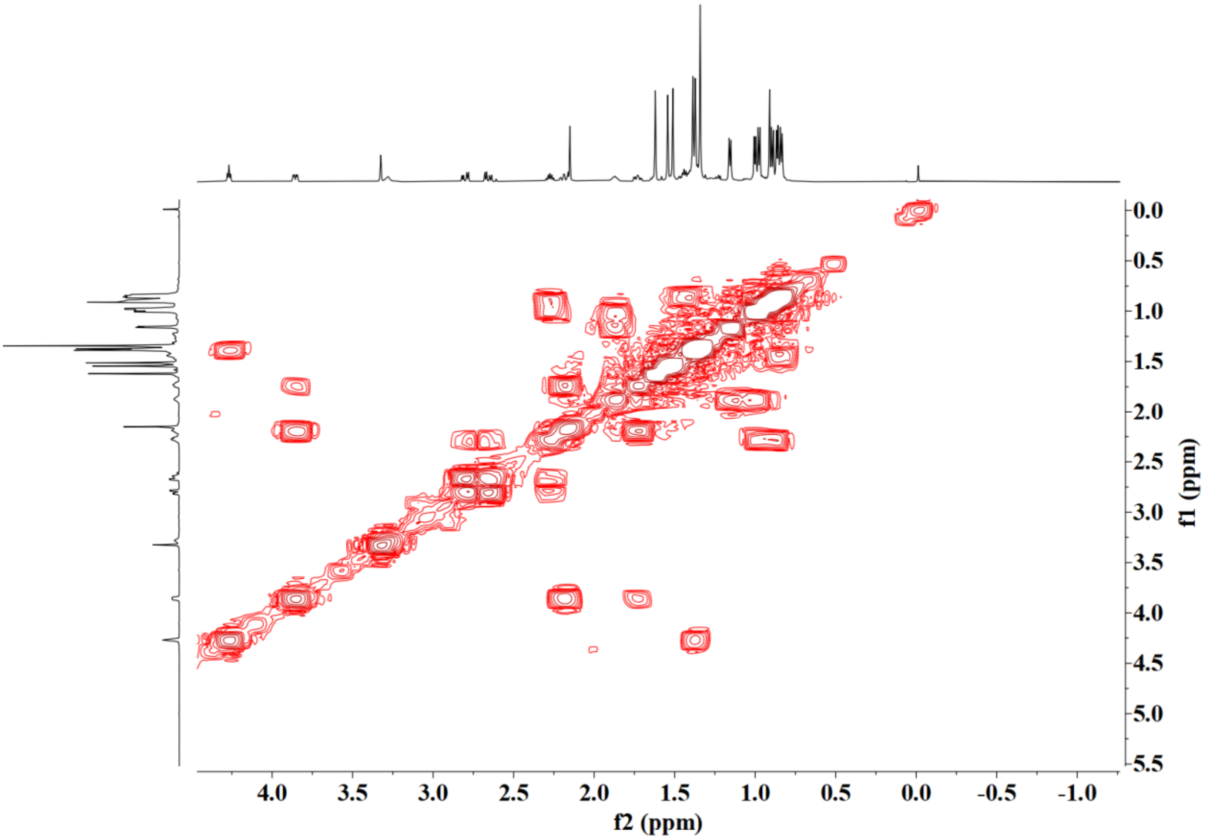

Fig. S15 HMBC spectrum of callistemontrimer B.

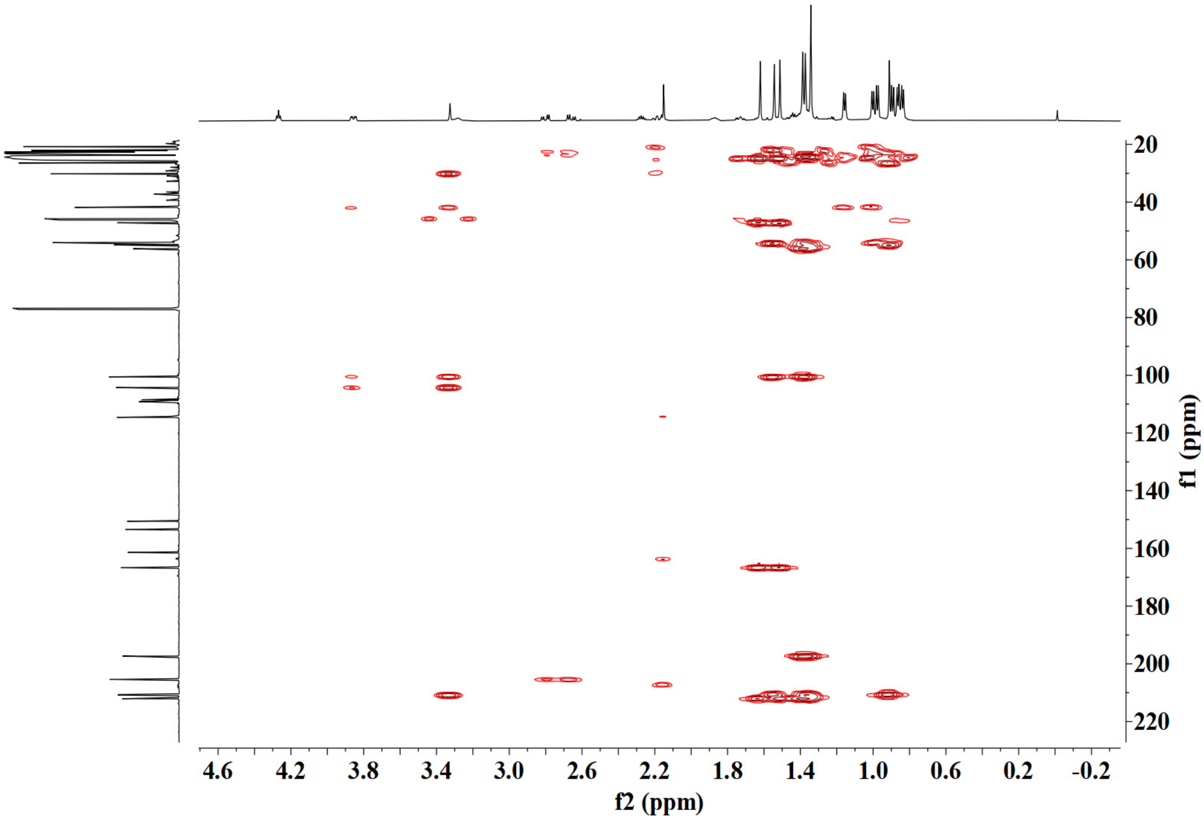

Fig. S16 ROESY spectrum of callistemontrimer B.

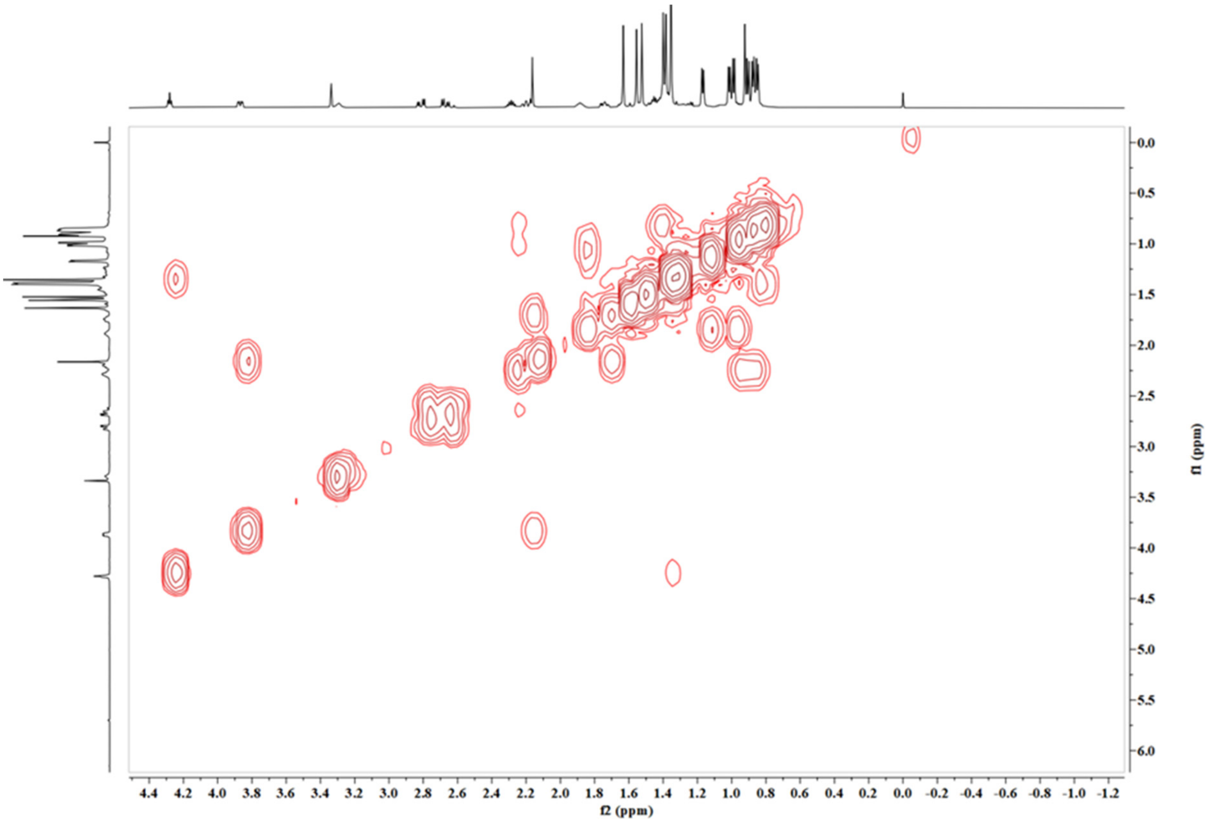

Fig. S17 Calculated ECD data of callistemontrimer B.

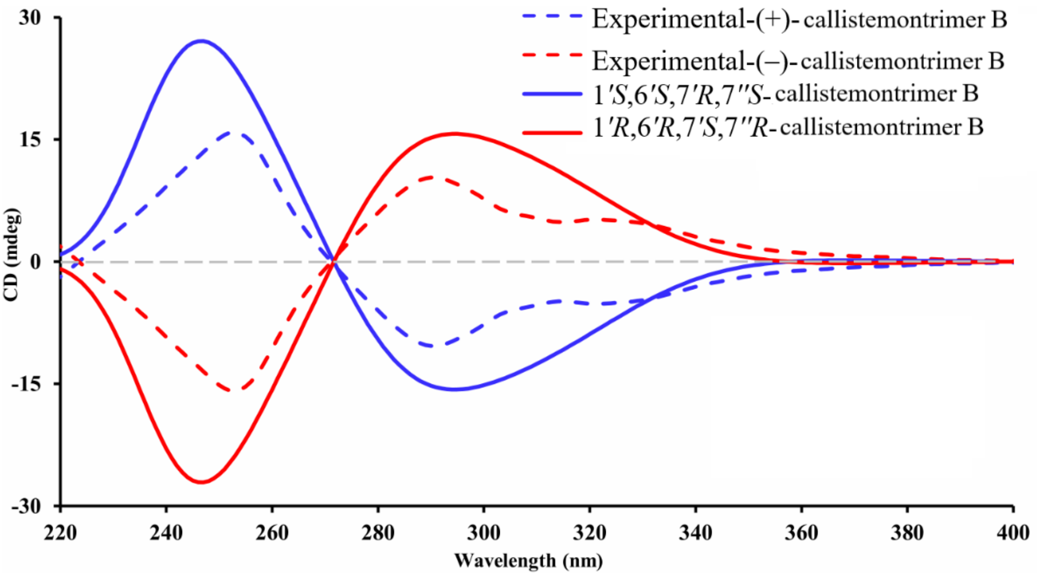

Fig. S18 Anti-HTNV activities of compounds.

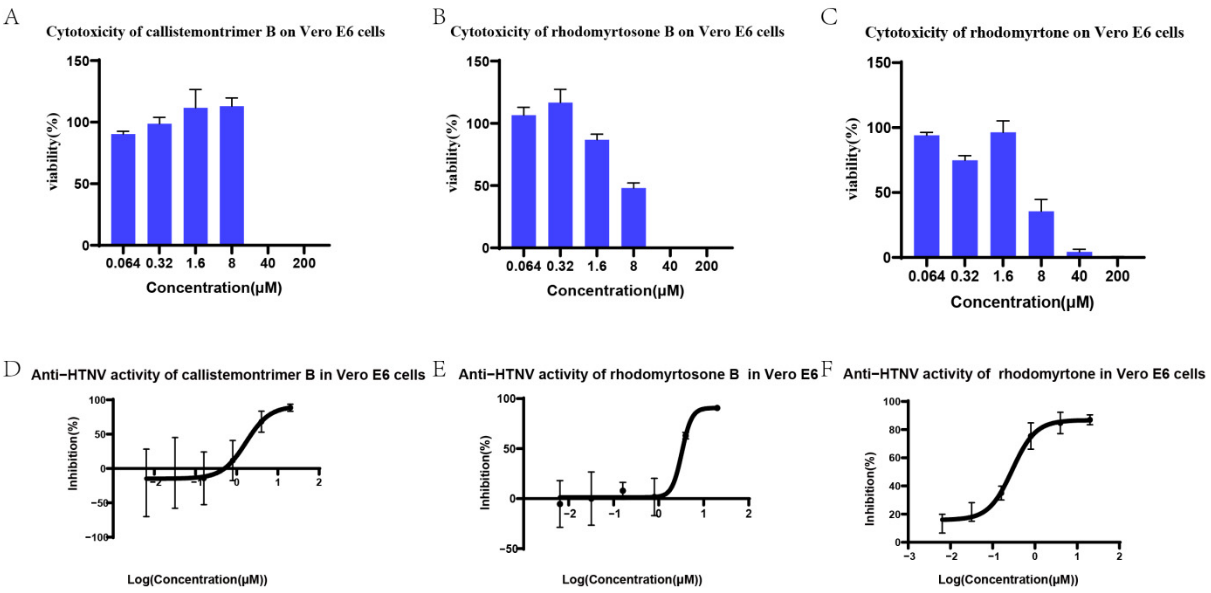

Antiviral activity of compounds. The cytotoxicity of callistemontrimer B, rhodomyrtosone B, and rhodomyrtone on Vero E6 (A, B and C) was determined using the MTT assay after co-incubation of the compounds with the cells for 3 days. Dose–response curves show quantitation of HTNV S segment RNA levels in Vero E6 cells (C, D and E) after HTNV treatment with gradient-diluted compounds for 5 days. All data represent means ± standard deviation (SD) for two independent replicate experiments.

Fig. S19 Visualization of the inhibitory effects of callistemontrimer A and ribavirin on HTNV replication.

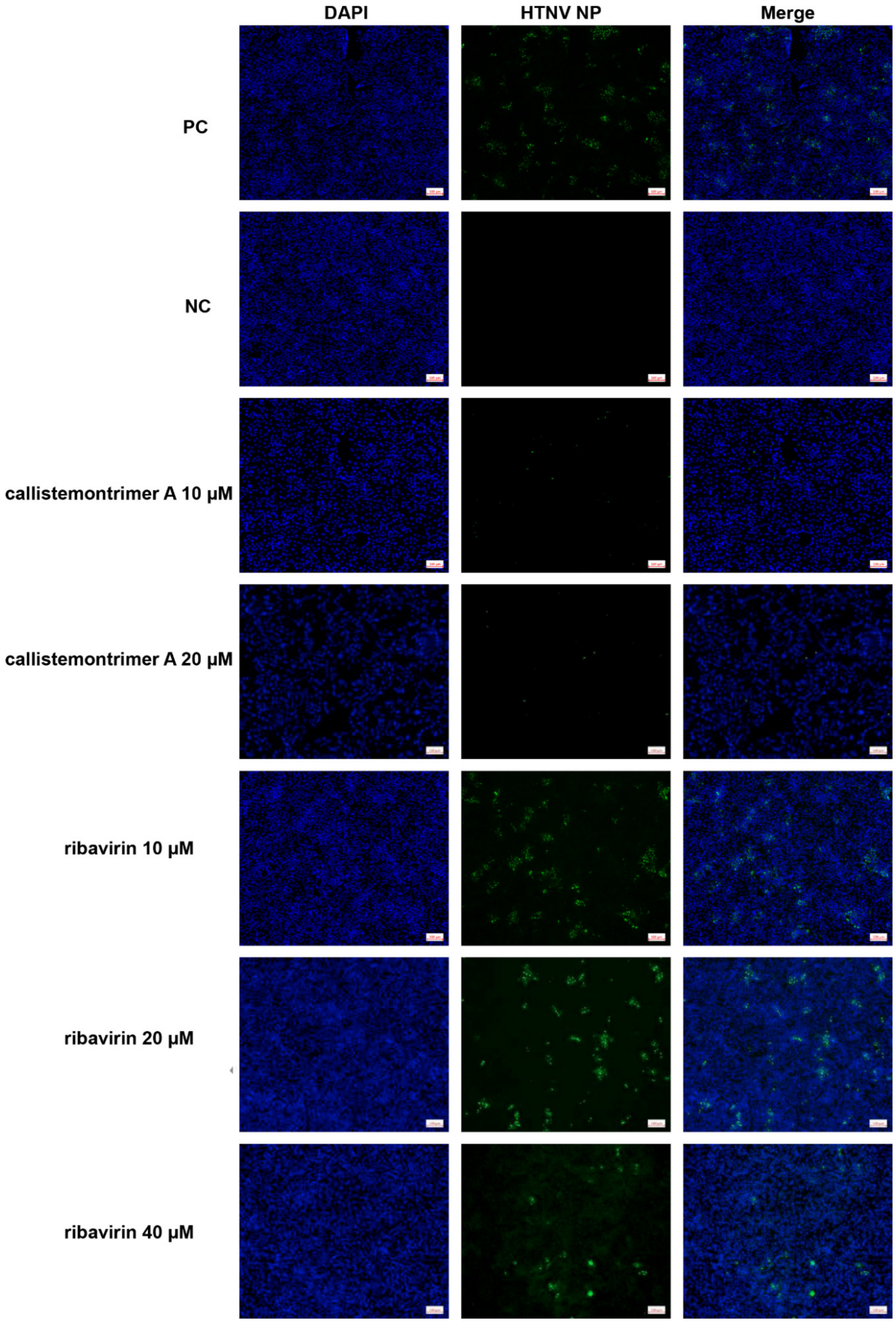

Inhibitory effects of callistemontrimer A and ribavirin on HTNV replication. Compounds of different concentrations were added after HTNV infection at an MOI of 1. At 48 h post-infection, the cells were fixed, permeabilized, and stained with an antibody against the HTNV nucleoprotein. Blue, cell nuclei; green, HTNV nucleoprotein.
